# Characterization and Quantification of Polyphenols and Triterpenoids in Thinned Young Fruits of Ten Pear Varieties by UPLC-Q TRAP-MS/MS

**DOI:** 10.3390/molecules24010159

**Published:** 2019-01-03

**Authors:** Liqiong Sun, Shutian Tao, Shaoling Zhang

**Affiliations:** Center of Pear Engineering Technology Research, State Key Laboratory of Crop Genetics and Germplasm Enhancement, Nanjing Agricultural University, Nanjing 210095, China; sarlign@163.com (L.S.); taost@njau.edu.cn (S.T.)

**Keywords:** thinned young pear, polyphenol, triterpenoid, identification, quantification, mass spectrometry, antioxidant

## Abstract

Large quantities of thinned young pears, a natural source of bioactive compounds, are abandoned as agricultural by-products in many orchards. Hence, ten thinned young pear varieties were systematically investigated in terms of their chemical composition and antioxidant potential. Through ultra-performance liquid chromatography coupled with electrospray ionization triple quadrupole mass spectrometry (UPLC-Q TRAP-MS/MS), 102 polyphenols and 16 triterpenoids were identified and individually quantified within a short time using multiple reaction monitoring (MRM). Subsequently, the antioxidant capacities of these pears were determined with DPPH assays, and the correlation between total antioxidant activity and each component was analyzed. The results indicated that the bioactive compound content and antioxidant capacity in thinned pears were considerably high. Regarding chemical composition, chlorogenic acid, quinic acid and arbutin were the primary polyphenols and ursolic acid was the predominant triterpenoid, whereas 27 polyphenolic compounds, especially chlorogenic acid and most of the flavan-3-ols, were the main antioxidants in young pears. These findings should provide a scientific basis for the further use of pear fruit by-products.

## 1. Introduction

Pear (*Pyrus* spp.) is one of the most widely produced fruits in the world and is cultivated in more than 50 countries. It is a dietary source of bioactive components such as polyphenols and triterpenic acids [1,2]. In the marketplace, pear fruit size and quality are extremely important for successful commercialization. For this reason, thinning of pear trees is a cultural practice often adopted in all orchards to remove excess and undesirable fruit. Thinning is one of the most effective measures to improve fruit size and quality at harvest and to balance the pear yield in the following year [3]. According to the data published by the United Nations Food and Agriculture Organization, in 2016, the total harvesting area and pear production worldwide was 1584.96 thousand hectares and 27.35 million tonnes, respectively. Hence, many thinned young pears are produced every year and abandoned as agricultural by-products, thereby usually generating large quantities of waste, which may affect the growth of fruit trees by increasing the acidity and disturbing the microbial community of the grove soil [4]. Recent studies have indicated that these agricultural by-products might provide an agricultural and food resource, because the content of bioactive substances in unripe pears in early stages of growth was significantly higher than in later stages [5]. Therefore, collecting and using these young pears rather than discarding them directly in fruit orchards may be both valuable and necessary.

The mature pear fruit is typically eaten fresh and is also an important material for both pharmaceutical and food applications, due to its nutritional and health promoting benefits, such as anti-inflammatory, antioxidant, antitussive, anti-diabetic, antimicrobial and diuretic activities [2,6]. Polyphenols, the major functional ingredients in pears, have already been studied in different pear varieties [7,8], and their health-promoting benefits have been demonstrated to be closely associated with strong antioxidant and anti-inflammatory properties [7]. In addition, triterpenoid compounds—which are well known for their beneficial health properties, such as their anti-inflammatory, antioxidant, antiviral and immunomodulatory activities [9]—have also been reported to be abundant in pears [1,2]. Ursolic, oleanolic and betulinic acids are the commonly reported compounds in mature pears. Nevertheless, the chemical composition characteristics in immature pears still require further study.

In recent years, ultra-performance liquid chromatography coupled with triple quadrupole mass spectrometry (UPLC-MS/MS) has been widely and successfully used for characterizing and quantifying many compounds in complex samples [10,11]. Especially, through use of the multiple reaction monitoring (MRM) scan mode, MS/MS detection could achieve high sensitivity and selectivity without establishing baseline separation of the target analytes, and a large array of compounds could be simultaneously determined in a very short time [12]. In addition, precursor and production ion monitoring could not only increase the specificity of detection but also aid in identifying the molecules. Consequently, we reasoned that UPLC-MS/MS might be an ideal strategy for the determination of multiple chemical profiles from pears.

Hence, in the present study, we developed an ultra-performance liquid chromatography coupled with electrospray ionization triple quadrupole mass spectrometry (UPLC-Q TRAP-MS/MS) system to characterize and quantify the bioactive components in ten varieties of thinned young pears. The high resolution of UPLC and the sensitivity of the MRM mode allowed us to simultaneously monitor 102 polyphenols within 15 min and to quantify 16 triterpenoids within another 10 min. The differences in the content of bioactive components among various pear varieties were assessed. Moreover, the antioxidant capacities of young pears in vitro were investigated with 1,1-diphenyl-2-picrylhydrazyl radical (DPPH) assays, and a correlation analysis between total antioxidant activity and the content of individual component was performed to identify potential bioactive compounds. This study might provide a scientific basis for further use of discarded thinned-young pears.

## 2. Results and Discussion

### 2.1. Identification of Polyphenol and Triterpenoid Composition

The characteristic phenolic and triterpene profiles in ten varieties of thinned young pears were investigated rapidly with high sensitivity through UPLC-Q TRAP-MS/MS coupled with information-dependent acquisition (IDA) method. The compounds were unambiguously or tentatively identified on the basis of comparison of retention time and mass spectral data with available standards and published data [7,8,13,14,15,16,17,18,19,20,21,22,23,24,25,26,27]. As presented in Table 1 and Figure S1, 102 polyphenloic compounds were rapidly separated from pear extracts within 15 min. The compounds belonged to different chemical classes: phenolic acids (28), phenolic glycosides (16), flavones (33) and flavan-3-ols (25). Besides, 16 triterpenoids were detected within another 10 min, and some of them were detected and characterized from pear species for the first time.

The [M − H]^−^ nominal mass and the characteristic fragment ion in negative mode (Table 1) were used for component identification. In accordance with previous studies, neutral loss of H_2_O molecules (18 Da) was frequently found for phenolic acids, such as quinic acid (peak 1) [13,14]. Direct loss of the sugar moiety was characteristically detected from the phenolic glycosides and flavones, such as arbutin (peak 3), roseoside (peak 23), rutin (peak 52), hyperoside (peak 55) and luteoloside (peak 62), and the fragmentation of all these compounds yielded aglycone ions [8,15,16]. The esterified compounds derived from two or three phenolic acids were easily distinguished on the basis of their typical fragmentation patterns, owing to the loss of phenolic acid residues; these compounds included caffeoylquinic acid (peaks 5, 13, 15 and 20), dicaffeoylquinic acid (peaks 61, 76, 83 and 92), *p*-coumaroylquinic acid (peaks 25 and 34) and feruloylquinic acid (peak 31) [13,14,16,17]. In addition, characteristic fragment ions of five arbutin conjugates, including two *p*-coumaroylarbutins (peaks 68 and 85) and three caffeoylarbutins (peaks 39, 42 and 57), were obtained by loss of hydroquinone (110 Da) and the arbutin moiety (254 Da) [18]. Regarding different polymeric procyanidins compounds (peaks 30, 37, 40, 44, etc.) formed from two to four catechin and epicatechin molecules, their identical fragmentation pathways may have occurred through loss of (epi)catechin residues or the retro-Diels-Alder fragment [13]. In particular, apart from the most common triterpenic compounds in pears, such as betulinic, oleanolic and ursolic acid (peaks 13ʹ, 14ʹ and 15ʹ), 13 other triterpenic acids were identified from young pear extracts, and their typical resulting product ions corresponded to the loss of H_2_O (18 Da), CO_2_ (44 Da) and CH_2_O (30 Da) [23,24,25,26,27].

### 2.2. Quantification of Polyphenols and Triterpenoids

**Figure 1:**
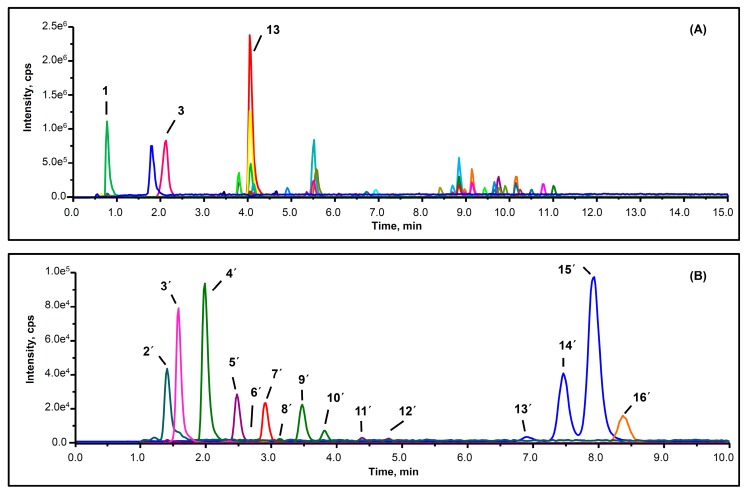
Representative UPLC-MS/MS chromatograms of thinned young pears in the multiple reaction monitoring (MRM) mode. (**A**) the polyphenolic profiles of ‘Hongqieli’ extract, (**B**) the triterpenoid profiles of ‘Yali’ extract.

Most compounds obtained showed a symmetrical peak shape and good resolution under the present chromatographic conditions (Figure 1 and Figure S2). Before sample analysis, the performance of the method was validated through a series of tests. The linearity, regression and linear ranges of 23 available compounds were obtained by using the external standard method and are listed in Table 2. All of the correlation coefficient values (R^2^ > 0.9995) indicated appropriate correlations between the concentrations of investigated compounds and their peak areas within the test ranges. The limits of detection (LODs) and limits of quantitation (LOQs) were also estimated on the basis of 3:1 and 10:1 signal-to-noise (S/N) ratios obtained with mixed standards containing the compounds at low concentration. The relative standard deviation (RSD) values for intra- and inter-day precision ranged from 0.43% to 1.12% and 1.47% to 3.26%, respectively, thus indicating acceptable precision of the method (Table S1). The RSD values of experimental repeatability were below 7.65% for the contents of all peaks, thus indicating that the method was repeatable (Tables S2 and S3). In addition, the sample stability RSD values were below 6.74% in 72 h, thus indicating that the analytes were stable (Tables S2 and S3). All results demonstrated that the developed method could provide a reliable, sensitive and stable means for the quantitative analysis of various pear varieties.

**Figure 2:**
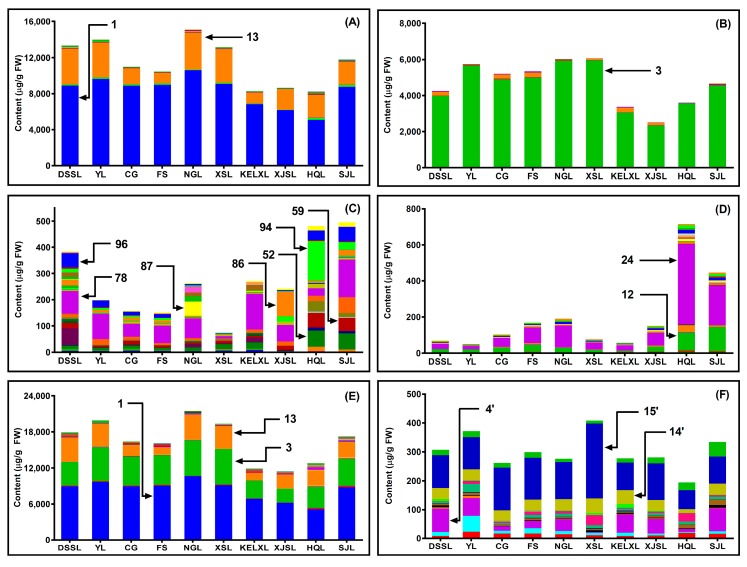
The content of chemical constituents in thinned young pears. (**A**) 28 phenolic acids, (**B**) 16 phenolic glycosides, (**C**) 33 flavones, (**D**) 25 flavan-3-ols and procyanidins, (**E**) total polyphenloic compounds, (**F**) 16 triterpenoids. (1) quinic acid; (3) arbutin; (12) (+)-catechin; (13) chlorogenic acid; (24) (−)-epicatechin; (52) rutin; (59) quercetin-3*-O-*glucoside; (78) isorhamnetin-3*-O-*rutinoside; (86) isorhamnetin-3*-O-*glucoside; (87) kaempferol-3*-O-*glucoside; (94) kaempferol-acylated-galactoside; (96) isorhamnetin-acylated-glucoside; (4′) pomolic acid isomer; (14′) oleanolic acid; (15′) ursolic acid.

Using the proposed UPLC-MS/MS method, the quantitative analysis of 10 pear varieties was performed through the external standard methods. The thinned young pears were found to be a valuable source of bioactive molecules (Figure 2 and Table 3). The total polyphenolic content ranged from 11.4 mg/g fresh weight (FW) in ‘Xinjiangsuanli’ to 21.4 mg/g FW in ‘Nanguoli’ sample, with an average of 16.4 mg/g FW, while the total triterpenoid content ranged from 193.0 μg/g FW in ‘Hongqieli’ to 403.1 μg/g FW in ‘Xiangshuili’, with an average of 298.7 μg/g FW. These concentrations in immature pears were clearly higher than those in many mature pears previously reported by other authors [13,28]. The chemical composition showed similar characteristics among different pear varieties (Figure 2). Quinic acid, arbutin and chlorogenic acid (peaks 1, 3 and 13) were the major polyphenolic constituents in various pear fruitlets, and pomolic acid analogue, oleanolic acid and ursolic acid (peaks 4′, 14′ and 15′) were the predominant triterpenic compounds. However, there were still many significant discrepancies among different pear varieties.

Phenolic acids are the most representative polyphenols in pears and other fruits [7,29], accounting for >64% of all the polyphenolic compounds in the investigated young pears (Table 3). Relatively higher total phenolic acid content was found in thinned ‘Nanguoli’, ‘Yali’, ‘Dangshansuli’ and ‘Xiangshuili’ (14,994.6, 13,905.9, 13,242.2 and 13,052.6 μg/g FW, respectively), whereas lower content was observed in ‘Hongqieli’, ‘Kuerlexiangli’ and ‘Xinjiangsuanli’ varieties (8123.9, 8172.6 and 8541.3 μg/g FW, respectively). The primary phenolic acid, quinic acid (peak 1), accounted for ~ 73% of the total phenolic acid content and ranged from 10,500.5 μg/g FW in ‘Nanguoli’ to 4991.7 μg/g FW in thinned ‘Hongqieli’. These values were in marked contrast to previously reported ranges of mature pears [1,7]. In published literature, little quinic acid (≤0.2 mg/100 g of dry matter) could be found in different anatomical parts of ripe pears [1]. These results suggest that, as the raw material of many secondary metabolic products, quinic acid would be largely exhausted during the process of pear fruit growth and development. The second predominant phenolic acid, chlorogenic acid (peak 13), accounted for approximately 24% of the total phenolic acid and varied from 4139.0 μg/g FW in ‘Nanguoli’ to 1162.7 μg/g FW in ‘Fengshui’. Although chlorogenic acid remains abundant in phenolic compounds during pear fruit development, the average concentration in thinned pears was ~34 times higher than that in fully ripe pears reported by others [13].

Arbutin (peak 3) was the most abundant phenolic glycoside compound, accounting for >90% of the total phenolic glycosides in young pears and ranging from 5905.9 μg/g FW in ‘Xiangshuili’ to 2285.6 μg/g FW in ‘Xinjiangsuanli’. The levels were clearly higher than those in ripe pears [1,2]. Accordingly, the abundant arbutin in young pears would be gradually metabolized to other compounds as pear fruits grow and mature. Besides, the content of arbutin and total phenolic glycoside in *Pyrus sinkiangensis* (‘Kuerlexiangli’ and ‘Xinjiangsuanli’) and *Pyrus communis* (‘Hongqieli’ and ‘Sanjili’) varieties were much lower than those of other species. This result was similar to the findings for phenolic acid and total polyphenol content, thus implying a possible relationship in the biosynthetic regulation of phenolic acids and phenolic glycosides in pears.

The total content of flavones in the investigated pear varieties (Figure 2C and Table 3) was clearly much lower than that of phenolic acids and phenolic glycosides, and it accounted for only ~ 1.6% of the total polyphenol content, because most of the flavone compounds are located in the pear peel but not the flesh [7,28]. Nonetheless, the samples differed distinctly in terms of flavone composition and content. As *P. communis* cultivars, the young fruits of ‘Sanjili’ and ‘Hongqieli’ obviously showed the highest total flavone content (492.2 and 478.1 μg/g FW, respectively), attributing to the abundance of rutin, quercetin-3-*O*-glucoside, isorhamnetin-3-*O*-rutinoside, kaempferol-acylated-galactoside and isorhamnetin-acylated-glucoside (peaks 52, 59, 78, 94 and 96). In contrast, the *Pyrus ussuriensis* sample obtained from ‘Xiangshuili’ showed the lowest flavone content, with a total value of 71.1 μg/g FW. Of the 33 flavones monitored in this study, only nine compounds were found in all the fruits tested, including rutin, quercetin-3-*O*-galactoside, quercetin-3-*O*-glucoside, Kaempferol-3-*O*-rutinoside, isorhamnetin-3-*O*-rhamnosylgalactoside, isorhamnetin-3-*O*-rutinoside, kaempferol-3-*O*-galactoside and isorhamnetin-3-*O*-glucoside (peaks 52, 55, 59, 73, 75, 78, 79 and 86). Those compounds are also the major flavonols in the peels of ripe pears, as reported by others [2,28]. As the distinguished flavone in pears [30], the highest content of isorhamnetin-3-*O*-rutinoside (peak 78) was 143.8 μg/g FW in ‘Sanjili’, whereas the lowest was only 9.1 μg/g FW in ‘Xiangshuili’. Meanwhile, some other distinguished flavones were also found in different varieties. The ‘Nanguoli’ sample was characterized by kaempferol-3-*O*-glucoside (peak 87) with an exceptionally high content value of 55.1 μg/g FW, while the ‘Xinjiangsuanli’ sample, which showed lower total flavone content, contained markedly higher amounts of isorhamnetin-3-*O-*glucoside (peak 86) with a value of 95.1 μg/g FW.

The analysis also revealed statistically significant discrepancies in the concentrations of flavan-3-ols and procyanidins among different pear varieties (Figure 2D and Table 3). Reflecting a species characteristic of *P. communis*, the total content of flavan-3-ols in immature fruits of ‘Hongqieli’ and ‘Sanjili’ (711.7 and 441.2 μg/g FW) was not only markedly higher than those in other investigated pear species but also obviously higher than those in mature fruits of *P. communis* cultivars reported by others [13]. This result was similar to the comparison of the flavone amounts. Among 25 flavan-3-ols in the 10 young pear varieties, (−)-epicatechin (peak 24) was predominant (ranging from 445.7 μg/g FW in ‘Hongqieli’ to 18.3 μg/g FW in ‘Yali’) and was followed by (+)-catechin (peak 12) (ranging from 134.3 μg/g FW in ‘Sanjili’ to 7.0 μg/g FW in ‘Kuerlexiangli’). 

Besides polyphenols, triterpenes are also important biologically active constituents of pears. Therefore, the content of ursolic, oleanolic and betulinic acid has often been used in quality evaluation indexes for pears and other-related products [2]. In this paper, besides these three compounds, 13 other triterpenes were investigated in pear fruits. Among 16 triterpene compounds in the tested pears, ursolic acid was predominant (peak 15′, ranging from 257.1 μg/g FW in ‘Xiangshuili’ to 66.7 μg/g FW in ‘Hongqieli’) and was followed by the pomolic acid isomer (peak 4′, ranging from 82.2 μg/g FW in ‘Dangshansuli’ to 2.9 μg/g FW in ‘Xiangshuili’) and oleanolic acid (peak 14′, ranging from 49.9 μg/g FW in ‘Xiangshuili’ to 13.5 μg/g FW in ‘Hongqieli’) (Figure 2F and Table 3). This result might aid in further study of the triterpene composition in mature pears and related products.

### 2.3. Antioxidant Capacity of Thinned Pears

To obtain an overall view of the potent health benefits of thinned pears, the antioxidant capacities of 10 varieties were measured through DPPH analysis and expressed as micromolar Trolox equivalent per gram fresh weight (μmol TE/g FW) (Table 4). Among these samples, ‘Hongqieli’ variety showed the strongest antioxidant potential (30.4 μmol TE/g FW) and was followed by ‘Xinjiangsuanli’, ‘Xiangshuili’ and ‘Nanguoli’ (22.5, 22.4 and 22.2 μmol TE/g FW, respectively). In contrast, ‘Kuerlexiangli’, ‘Cuiguan’ and ‘Fengshui’ (10.1, 12.4 and 15.6 μmol TE/g FW, respectively) had the lowest antioxidant activity. Owing to the diversity in the content of chemical compounds contained in different varieties, the coefficient of variance for total antioxidant activity was 29.4%. Nevertheless, the antioxidant capacity of the thinned young pears was significantly higher than that reported by other authors for mature pears [1], in agreement with previous literature [5]. Recently, some studies proved a highly significant linear correlation between antioxidant capacity and polyphenol content in fruits and vegetables [31]. Therefore, similar strong antioxidant activity of ‘Xiangshuili’ and ‘Nanguoli’ might be attributed to their abundant polyphenols, whereas the moderate antioxidant potential of ‘Kuerlexiangli’ might be also influenced by its low polyphenol content. However, the fruitlets of ‘Hongqieli’ and ‘Sanjili’, which were poor in total polyphenols but rich in flavan-3-ols, still had high antioxidant capacity, thus indicating that the free radical scavenging activity of flavan-3-ols might be much stronger than those of other polyphenols. In addition, of interest was the finding that ‘Xinjiangsuanli’, in which the content of most polyphenolic compounds was lower than those in other varieties except isorhamnetin-3-*O*-glucoside (peak 86), also showed strong antioxidant potential. One of the reasons for this result might be that some other biologically active compounds undetected by the current method might contribute to the free radical scavenging activity of the ‘Xinjiangsuanli’ sample.

### 2.4. Hierarchical Clustering Analysis

**Figure 3:**
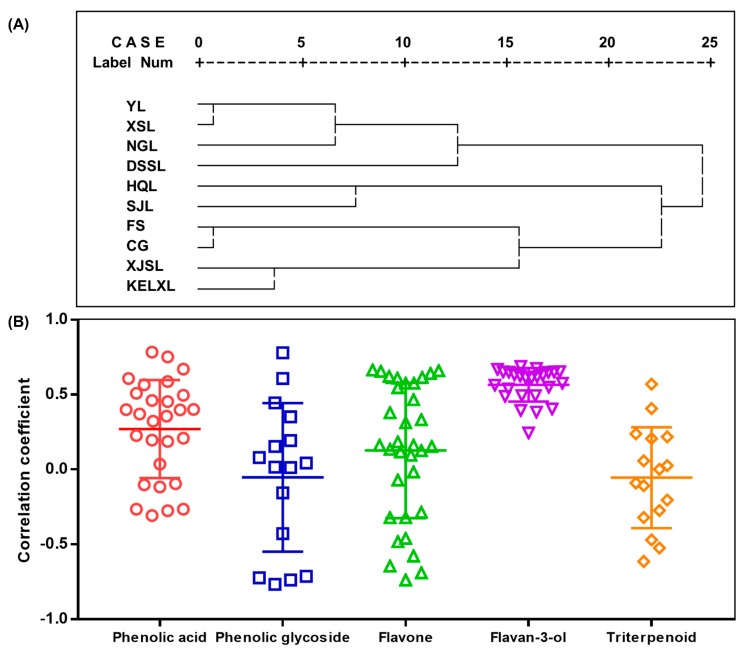
Hierarchical clustering analysis of pear samples (**A**) and the spectrum–effect relationship between total antioxidant activity and individual chemical compound (**B**).

Hierarchical clustering analysis (HCA) was performed to discriminate different pear samples based on the chemical constituents and antioxidant activity. The result of HCA is shown in Figure 3A. It was obvious that the samples could be classified into three clusters: two *Pyrus bretschneideri* Rehd. (‘Yali’ and ‘Dangshansuli’) and two *P. ussuriensis* Maxim. (‘Xiangshuili’ and ‘Nanguoli’) in cluster 1, two *P. communis* L. (‘Hongqieli’ and ‘Sanjili’) in cluster 2, two *Pyrus pyrifolia* Nakai (‘Fengshui’ and ‘Cuiguan’) and two *P. sinkiangensis* Yu (‘Kuerlexiangli’ and ‘Xinjiangsuanli’) in cluster 3. In cluster 3, *P. pyrifolia* Nakai and *P. sinkiangensis* Yu samples were distinctly divided into two groups. There were relatively small differences among the samples from the same pear species. The results were broadly consistent with the cultivar classification system of pear fruit [32] and showed that different pear species could be clearly distinguished according to their chemical and antioxidant characteristics. 

### 2.5. Correlation Analysis

A correlation analysis between total antioxidant activity and each component content was carried out (Figure 3B and Table S4), which further demonstrated that thinned pears containing higher levels of bioactive compounds had more potent health benefits. The results indicated that predominant quinic acid and arbutin had a weak influence on the total activity, whereas chlorogenic acid, the other primary phenolic compound, exhibited a noticeable correlation (R = 0.566). This result was quite similar to those reported by other authors [33,34]. Consequently, the strong antioxidant activity of ‘Dangshansuli’, ‘Yali’, ‘Xiangshuili’ and ‘Nanguoli’ might be attributed to the higher content of chlorogenic acid, whereas the unsatisfactory activity of ‘Kuerlexiangli’, ‘Cuiguan’ and ‘Fengshui’ might also be ascribed to the lower content of this compound. In addition, there were 27 polyphenolic compounds, including four phenolic acids (peaks 16, 20, 25 and 34), two phenolic glycosides (peaks 26 and 102), seven flavones (peaks 50, 55, 65, 73, 74, 80 and 94) and 14 flavan-3-ols (peaks 4, 17, 18, 19, 24, 29, 30, 33, 40, 53, 60, 66, 70 and 84), that showed a marked positive correlation (R > 0.6) with the antioxidant activity of the pear extracts, and these compounds might be the principal antioxidant contributors. These results provide further evidence that young pears may be a natural source of biological antioxidants in the pharmaceutical, cosmetic and food industries. In particular, all of flavan-3-ol compounds abundant in *P. communis* cultivars showed a significant positive correlation with the total antioxidant activity, thus indicating that the young fruits of *P. communis* might have advantages in antioxidant related industries. However, there were still eight compounds, including four phenolic glycosides (peaks 42, 57, 68 and 85), four flavones (peaks 38, 41, 43 and 101) and two triterpenic acids (peaks 1ʹ and 14ʹ), showing an obvious negative correlation (R < −0.5) with the total activity. All of the four phenolic glycosides were arbutin derivatives according to their aglycon. In published literature, it was reported that the DPPH scavenging capacity of oleanolic acid was significantly lower than phenolic compounds, such as chlorogenic acid and procyanidin compound [35]. In pear extracts, most triterpenoid constituents except corosolic acid showed a weak contribution to the total antioxidant activity, perhaps due to a similar molecular skeleton. Nevertheless, other health benefits of triterpenoids have been demonstrated by many researchers [9,26,36].

## 3. Materials and Methods

### 3.1. Reagents and Standards

HPLC-grade acetonitrile was purchased from Merck (Darmstadt, Germany). HPLC-grade formic acid, DPPH and 6-hydroxy-2,5,7,8-tetramethylchroman-2-carboxylic acid (Trolox) were purchased from Sigma-Aldrich (Steinheim, Germany). HPLC-grade water was purified with a Millipore Water Purification System (Bedford, MA, USA). The standards arbutin, (+)-catechin, (−)-epicatechin, neochlorogenic acid, cryptochlorogenic acid, chlorogenic acid, rutin, oleanolic, ursolic, *p*-coumaric, caffeic and quinic acid were purchased from the National Institute for the Control of Pharmaceutical and Biological Products (Beijing, China). Luteoloside, kaempferol-3-*O*-rutinoside, isorhamnetin-3-*O*-rutinoside, isochlorogenic acid A, isochlorogenic acid B, isochlorogenic acid C and hyperoside were bought from Beijing Solarbio Science and Technology Co., Ltd. (Beijing, China). Betulinic, pomolic, corosolic and maslinic acid were purchased from Wuhan ChemFaces Biochemical Co., Ltd. (Wuhan, China). The purity of each reference standard was determined to be higher than 98% by HPLC.

### 3.2. Materials

**Figure 4:**
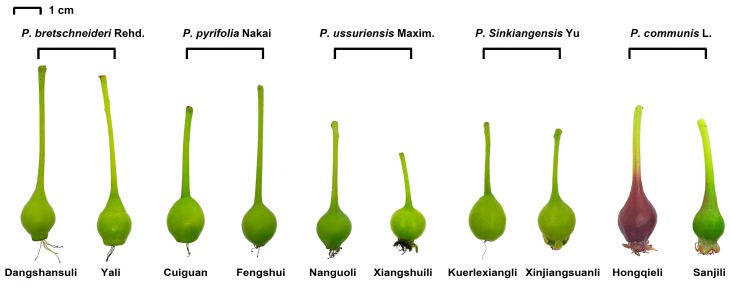
Visual appearance of thinned young fruits from ten pear varieties at 20 days after full bloom.

According to the classification system of pears from previous literature [32], 10 pear varieties from five typical *Pyrus* species were selected and their fruitlets were hand-thinned about 20 days after full bloom at pear orchards in various regions of China (Figure 4). For two *P. pyrifolia* Nakai, thinned fruits of ‘Fengshui’ (FS) and ‘Cuiguan’ (CG) were obtained individually from the pear orchard of Nanjing Agricultural University (Jiangsu, China). For two *P. bretschneideri* Rehd., thinned fruits of ‘Dangshansuli’ (DSSL) were obtained from a commercial orchard in Gaoyou (Jiangsu, China), and those of ‘Yali’ (YL) were obtained from the Shijiazhuang Fruit Institute (Hebei, China). For two *P. ussuriensis* Maxim., thinned fruits of ‘Nanguoli’ (NGL) and ‘Xiangshuili’ (XSL) were obtained individually from the Liaoning Fruit Institute (Liaoning, China). For two *P. communis* L., thinned fruits of ‘Starkrimson’ (Hongqieli, HQL) and ‘Docteur Jules Guyot’ (Sanjili, SJL) were obtained individually from the Yantai Agricultural Research Academy (Shandong, China). For two *P. sinkiangensis* Yu, thinned fruits of ‘Kuerlexiangli’ (KELXL) and ‘Xinjiangsuanli’ (XJSL) were obtained individually from the Xiangli Fruit Institute (Xinjiang, China).

In the course of the measurements, five trees for each variety, which were randomly located and had comparable growth vigor and tree age, were selected to ensure that the samples were representative. For each variety, 150 fruitlets (30 samples per tree) of similar size and with an absence of defects were randomly and manually picked. After collection, the fruits were immediately transferred to the laboratory, washed with water, cut and directly frozen in liquid nitrogen, then crushed to a homogeneous powder with a laboratory mill in liquid nitrogen (Jingxin Industrial Development Co., Shanghai, China). The powders were kept at −80 °C until analysis.

### 3.3. Extract Preparation

Some studies have proved that an aqueous alcohol system and sonication could improve the extraction of polyphenolics [37]. The pear extracts were prepared as described in previous literature [38], with some modifications. Briefly, the powdered frozen pear samples were extracted with mixed methanol/water (80:20, *v*/*v*) and vortexed for 30 s, then sonicated (300 W, 50 kHz) for 30 min at 30 °C. The ratio of frozen powders and solvents was 1 g per 25 mL. The sample tube was shaken at 5-min intervals during sonication to resuspend the sample. This method has been demonstrated to be adequate for complete extraction. Next, the slurry was centrifuged at 20,000× *g* (Microfuge 20, Beckman Coulter Inc., CA, USA) for 15 min, and the supernatant was filtered through a 0.22 µm membrane and kept at 4 °C prior to analysis.

### 3.4. UPLC-Q TRAP-MS/MS Analysis of Polyphenols and Triterpenoids

A Shimadzu UPLC system (Shimadzu, Kyoto, Japan), interfaced with an AB Sciex 4500 Q trap mass spectrometer (AB Sciex, Foster City, CA, USA), installed with an electrospray ionization interface and a six-port/two-channel valve, was used for UPLC-Q TRAP-MS/MS analysis. The Shimadzu UPLC system consisted of two LC-20ADXR solvent delivery units, a Shimadzu LC-20AD pump, a SIL-20ACXR autosampler, a CTO-20AC column oven, a DGU-20A3R degasser, and a CBM-20A controller. AB Sciex Analyst Software Package Version 1.6.3 was applied to control the entire system, data acquisition and processing.

#### 3.4.1. UPLC Analysis

Chromatographic separation of bioactive components was accomplished with a Waters Acquity UPLC HSS T3 C18 column (2.1 mm × 100 mm, 1.8 μm, Milford, MA, USA) [11]. The temperature of the sample was set to 4°C, and the column temperature was maintained at 30 °C. The injection volume of the auto-sampler was set at 1 μL. For polyphenol composition analysis, the mobile phase consisted of 0.1% (*v*/*v*) aqueous formic acid (A) and acetonitrile (B) at a flow rate of 0.4 mL/min. The gradient elution program was as follows: 0–3 min, 1%–15% B; 3–11 min, 15%–40% B; 11–13 min, 40% B; 13–13.1 min, 40%–1% B; 13.1–15 min, 1% B. Before triterpenoid analysis, most pear extract solutions mentioned above were diluted 10 times with mixed methanol/water (80:20, *v*/*v*), whereas the extracted sample was diluted four times for ‘Hongqieli’ and 20 times for ‘Xiangshuili’ individually. Chromatographic analysis was then carried out through isocratic elution with methanol/water/ammonium acetate (81:19:0.1, *v*/*v*) at a flow rate of 0.4 mL/min.

#### 3.4.2. Identification of Bioactive Compounds in IDA Mode

The mass spectrometer was operated using enhanced MS scanning and enhanced product ion scanning in negative ion mode to identify polyphenolic and triterpenoid compounds in the pears. Enhanced product ion scans were triggered from the IDA method. The criterion for IDA was set for the two most intense ions after each dynamic survey scan spectrum with an intensity threshold of 5000 cps, and former target ions were never excluded during scanning. The parameters for enhanced product ion scanning were as follows: declustering potential (DP), 100 V; collision energy (CE), 35 eV; and collision energy spread (CES), 15 V. Nitrogen was used as the nebulizer, curtain, heater and collision gas. The ion source temperature (TEM) was raised to 500 °C, and the ion-spray voltages (IS) were maintained at 4500 V in negative ion mode. The gas settings were as follows: nebulizer gas (GS1) at 50 psi, heater (GS2) at 50 psi, curtain gas (CUR) at 30 psi and collision gas (CAD) at high level. The dwell time was fixed at 20 ms for each ion transition. Fragments formed in the enhanced product ion scans were detected in the mass range of *m*/*z* 100–1500 by using dynamic fill and a scan rate of 4000 Da/s.

#### 3.4.3. Quantification of Bioactive Compounds in MRM Mode

For quantification of bioactive components, mass spectrometric analysis was performed in negative mode by using electrospray ionization. The peak areas of all analytes were acquired in MRM mode for quantification. Optimization of MS parameters for each selected mass transition was carried out through direct infusion of a mixed standard solution and is summarized in Table S1. The other operating parameters were as follows: TEM, 500 °C; IS, 4500 V; GS1, 50 psi; GS2, 50 psi; and CUR, 30 psi. Then, calibration curves were determined experimentally for available standards. Because of a lack of standards for some tentatively identified compounds, their Q1 and Q3 were both set as quasi-molecular ions in MRM mode, and the DP values were optimized at 70, 100, 135, and 160 V. Their calibration curves were determined with some other available standards. Caffeic acid derivatives were expressed as caffeic acid, *p*-coumaric acid derivatives were expressed as *p*-coumaric acid, procyanidins were expressed as (−)-epicatechin, luteolin derivatives were expressed as luteoloside, isorhamnetin derivatives were expressed as isorhamnetin-3-*O*-rutinoside, kaempferol derivatives were expressed as kaempferol-3-*O*-rutinoside, quercetin derivatives and other flavonoid glycosides were expressed as rutin, and triterpenic acids were expressed as ursolic acid. All determinations were performed in triplicate.

#### 3.4.4. Validation of Methodology

The analytical performance of the methodology used for the quantification of bioactive compounds was investigated, including linearity, LOQs, LODs, stability, precision and repeatability. Serial calibration samples were prepared in methanol/water (80:20, *v*/*v*). The linearity was investigated with more than six concentration levels for each analyte, and each concentration was measured in triplicate. LOQs and LODs corresponded to the concentrations at S/N ratios of ~10 and 3, respectively.

To confirm instrument precision, the intra- and inter-day variations on the basis of relative standard deviations were assessed by using the calibration sample at a moderate concentration. For intra-day variability assays, the sample was measured in six replicates within one day, whereas the sample was examined in triplicate per day for three consecutive days to conduct inter-day assays.

Method repeatability was evaluated with injections of six extracts prepared independently from four selected samples (‘Dangshansuli’, ‘Nanguoli’, ‘Hongqieli’ and ‘Sanjili’) for polyphenol analysis and from two selected samples (‘Yali’ and ‘Nanguoli’) for triterpenoid analysis. Moreover, the tested sample solution was deposited at 4 °C, and the stability study of the pear extract was assessed at different time intervals (0, 12, 24, 48, and 72 h).

### 3.5. Analysis of Antioxidant Capacity

The antioxidant capacities of thinned young pears were measured through bleaching of the DPPH radical, as reported previously [2], with some modifications. Briefly, fresh DPPH stock solution was prepared by dissolving 19.7 mg DPPH in 100 mL of methanol and was then sealed and stored at 4 °C. One milliliter of different concentrations of pear extracts was mixed with 1 mL of DPPH solution. The mixture was shaken vigorously and incubated in the dark for 30 min at 37 °C, and the absorbance was then measured at 517 nm. All determinations were performed in triplicate. 

### 3.6. Statistical Analysis

Data are shown as the means of three independent determinations ± standard deviation. Hierarchical clustering analysis of ten pears was performed using the Statistical Package for the Social Science (SPSS) software version 22.0 (SPSS, Chicago, IL, USA) and the data was standardized before analysis. Multivariate correlation analysis was carried out in SPSS statistics software for evaluation of the spectrum–effect relationships between the content of each component and the total antioxidant capacity in thinned pears.

## 4. Conclusions

The aim of this study was to investigate the chemical composition and antioxidant potential of thinned pears. From five typical *Pyrus* species, ten pear varieties were selected. Then, 102 polyphenols and 16 triterpenoids were identified from these thinned young fruits through UPLC-Q TRAP-MS/MS in IDA mode, and detailed quantification of these compounds was accomplished within a short time by UPLC-MS/MS in MRM mode. To the best of our knowledge, this is the first report on simultaneous qualitative and quantitative analysis of 16 triterpenoid compounds in pears. According to the identified components, the most abundant polyphenolic constituents present were chlorogenic acid, quinic acid and arbutin, and ursolic acid was the dominant triterpenoid in young pears. Subsequently, the antioxidant capacities of these young pears were determined with DPPH assays, and a correlation analysis between total antioxidant activity and each component was performed. Twenty-seven polyphenolic compounds, especially chlorogenic acid and most of the flavan-3-ols, were found to be the principal antioxidant contributors. In addition, the bioactive compound content and antioxidant capacity of thinned pears were observed to be significantly higher in young pears than mature pears. These results may draw special attention to the potential health benefits of discarded thinned young pears as a natural source of bioactive components such as polyphenolics and triterpenoids. The health benefits of these fruit by-products are also promising and should be investigated further.

## Figures and Tables

**Table 1:** Characterization of chemical compounds in young pear fruits by ultra-performance liquid chromatography coupled with electrospray ionization triple quadrupole mass spectrometry (UPLC-Q TRAP-MS/MS).

Peak NO.	Rt (min)	Formula	[M − H]^−^ (*m*/*z*)	MS/MS Fragments (*m*/*z*)	Tentative Identification
***Polyphenol***					
1	0.81	C_7_H_12_O_6_	191.1	172.9[M − H − H_2_O]^−^	Quinic acid ^a^
2	1.76	C_9_H_8_O_2_	147.0	129.0[M − H − H_2_O]^−^	Cinnamic acid isomer
3	2.06	C_12_H_16_O_7_	317.1 ^#^	271.2[M − H]^−^, 160.9[M − H − C_6_H_6_O_2_]^−^, 109.0[M − H − Glc]^−^	Arbutin ^a^
4	2.77	C_30_H_24_O_12_	575.3	449.0[M − H − C_6_H_6_O_3_]^−^, 285.2[M − H − C_15_H_14_O_6_]^−^	A-type procyanidin dimer
5	3.19	C_16_H_18_O_9_	353.3	191.1[M − H − caffeoyl]^−^, 179.2[M − H − quinoyl]^−^	4*-O-*Caffeoylquinic acid (Cryptochlorogenic acid) ^a^
6	3.37	C_15_H_20_O_9_	343.2	181.0[M − H − hexose]^−^	Dihydro-caffeoyl*-O-*hexoside
7	3.48	C_30_H_26_O_12_	577.3	425.2[M − H − C_8_H_8_O_3_]^−^, 288.9[M − H − C_15_H_12_O_6_]^−^	B-type procyanidin dimer
8	3.51	C_15_H_20_O_8_	327.2	147.1[M − H − hexose − H_2_O]^−^	Hydroxyphenylpropionic acid*-O-*hexoside
9	3.76	C_15_H_20_O_10_	359.3	197.1[M − H − hexose]^−^	Syringic acid*-O-*hexoside
10	3.84	C_12_H_14_O_6_	253.1	135.1[M − H − C_3_H_6_O_2_ − CO_2_]^−^	Caffeoylglycerol
11	3.88	C_15_H_20_O_9_	343.2	181.0[M − H − hexose]^−^	Dihydro-caffeoyl*-O-*hexoside
12	4.11	C_15_H_14_O_6_	289.1	245.3[M − H − CO_2_]^−^, 202.9[M − H − H_2_O − C_3_O_2_]^−^	(+)-Catechin ^a^
13	4.13	C_16_H_18_O_9_	353.3	191.1[M − H − caffeoyl]^−^, 161.1[M − H − quinoyl − H_2_O]^−^	3*-O-*Caffeoylquinic acid (Chlorogenic acid) ^a^
14	4.31	C_45_H_38_O_18_	865.3	577.1[M − H − C_15_H_12_O_6_]^−^, 407.1[M − H − C_15_H_12_O_6_ − C_8_H_8_O_3_ − H_2_O]^−^, 289.0[M − H − 2C_15_H_12_O_6_]^−^	B-type procyanidin trimer
15	4.38	C_16_H_18_O_9_	353.3	191.1[M − H − caffeoyl]^−^, 173.0[M − H − caffeoyl − H_2_O]^−^	5*-O-*Caffeoylquinic acid (Neochlorogenic acid) ^a^
16	4.64	C_22_H_18_O_10_	441.3	325.1[M − H − C_4_H_4_O_4_]^−^, 163.0[M − H − C_4_H_4_O_4_ − caffeoyl]^−^, 118.9[*p*-coumaric acid − H − CO_2_]^−^	*p*-Coumaroylcaffeoyl malate
17	4.89	C_30_H_26_O_12_	577.3	425.1[M − H − C_8_H_8_O_3_]^−^, 407.0[M − H − C_8_H_8_O_3_ − H_2_O]^−^, 339.0[M − H − C_8_H_8_O_3_ − H_2_O − C_3_O_2_]^−^, 289.1[M − H − C_15_H_12_O_6_]^−^, 245.0[M − H − C_15_H_12_O_6_ − CO_2_]^−^	B-type procyanidin dimer
18	4.98	C_45_H_38_O_18_	865.3	577.0[M − H − C_15_H_12_O_6_]^−^, 287.1[M − H − C_15_H_12_O_6_ − C_15_H_14_O_6_]^−^	B-type procyanidin trimer
19	5.07	C_45_H_36_O_18_	863.3	573.3[M − H − C_15_H_14_O_6_]^−^, 289.2[M − H − C_15_H_12_O_6_ − C_15_H_10_O_6_]^−^	A-type procyanidin trimer
20	5.28	C_16_H_18_O_9_	353.3	191.1[M − H − caffeoyl]^−^	1*-O-*Caffeoylquinic acid
21	5.30	C_15_H_18_O_9_	341.1	178.9[M − H − hexose]^−^	Caffeoyl*-O-*hexoside
22	5.44	C_16_H_16_O_8_	335.3	179.1[M − H − shikimoyl]^−^	Caffeoylshikimic acid
23	5.45	C_19_H_30_O_8_	431.2 ^#^	385.0[M − H]^−^, 223.1[M − H − Glc]^−^	Roseoside
24	5.48	C_15_H_14_O_6_	289.1	245.0[M − H − CO_2_]^−^, 202.9[M − H − H_2_O − C_3_O_2_]^−^	(−)-Epicatechin ^a^
25	5.53	C_16_H_18_O_8_	337.3	190.9[M − H − *p*-coumaroyl]^−^, 163.2 [M − H − quinoyl]^−^	4-*p*-Coumaroylquinic acid
26	5.57	C_15_H_20_O_10_	359.3	197.0[M − H − hexose]^−^, 160.9[M − H − hexose − 2H_2_O]^−^	Syringic acid*-O-*hexoside
27	5.64	C_45_H_36_O_18_	863.3	711.3[M − H − C_8_H_8_O_3_]^−^, 573.0[M − H − C_15_H_14_O_6_]^−^, 289.0[M − H − C_15_H_12_O_6_ − C_15_H_10_O_6_]^−^	A-type procyanidin trimer
28	6.05	C_16_H_16_O_8_	335.3	179.0[M − H − shikimoyl]^−^, 135.1 [M − H − shikimoyl − CO_2_]^−^	Caffeoylshikimic acid
29	6.49	C_45_H_36_O_18_	863.3	711.1[M − H − C_8_H_8_O_3_]^−^, 573.0[M − H − C_15_H_14_O_6_]^−^, 451.2[M − H − C_15_H_12_O_6_ − C_7_H_8_O_2_]^−^, 289.2[M − H − C_15_H_12_O_6_ − C_15_H_10_O_6_]^−^	A-type procyanidin trimer
30	6.54	C_30_H_24_O_12_	575.3	285.3[M − H − C_15_H_14_O_6_]^−^	A-type procyanidin dimer
31	6.69	C_17_H_20_O_9_	367.4	191.0[M − H − feruloyl]^−^, 193.2[M − H − quinoyl]^−^	3*-O-*Feruloylquinic acid
32	6.71	C_19_H_16_O_12_	435.3	273.0[M − H − caffeoyl]^−^, 205.0[M − H − caffeoylmalonyl]^−^	Caffeoyl-malonyl-methylcitric aci
33	7.01	C_45_H_38_O_18_	865.3	577.1[M − H − C_15_H_12_O_6_]^−^, 451.1[M − H − C_15_H_12_O_6_ − C_6_H_6_O_3_]^−^, 407.1[M − H − C_15_H_12_O_6_ − C_8_H_8_O_3_ − H_2_O]^−^, 289.0[M − H − 2C_15_H_12_O_6_]^−^	B-type procyanidin trimer
34	7.24	C_16_H_18_O_8_	337.3	190.9[M − H − *p*-coumaroyl]^−^, 163.2 [M − H − quinoyl]^−^	5-*p*-Coumaroylquinic acid
35	7.35	C_30_H_26_O_12_	577.3	289.3[M − H − C_15_H_12_O_6_]^−^	B-type procyanidin dimer
36	7.40	C_17_H_20_O_9_	367.4	205.1[M − H − caffeoyl]^−^	4*-O-*Caffeoylquinic acid methyl ester
37	7.58	C_30_H_24_O_12_	575.3	285.2[M − H − C_15_H_14_O_6_]^−^	A-type procyanidin dimer
38	7.61	C_32_H_38_O_20_	741.4	301.1[M − H − Xyl-Rha-Gal]^−^	Quercetin-3*-O-*xylosylrhamnosylglucoside
39	7.84	C_21_H_22_O_10_	433.3	323.0[M − H − C_6_H_6_O_2_]^−^, 161.1[M − H − arbutin − H_2_O]^−^	Caffeoylarbutin
40	7.89	C_60_H_50_O_24_	1153.3	739.1[M − H − C_15_H_12_O_6_ − C_6_H_6_O_2_]^−^, 449.2[C_30_H_26_O_12_ − H − H_2_O − C_6_H_6_O_2_]^−^, 287.1[C_30_H_26_O_12_ − H − C_15_H_14_O_6_]^−^	B-type procyanidin tetramer
41	8.04	C_24_H_24_O_13_	519.4	315.3[M − H − acetyl-hexose]^−^	Isorhamnetin-acylated-hexoside
42	8.05	C_21_H_22_O_10_	433.3	323.0[M − H − C_6_H_6_O_2_]^−^, 178.9[M − H − arbutin]^−^, 160.9[M − H − arbutin − H_2_O]^−^, 133.1[M − H − arbutin − H_2_O − CO]^−^	Caffeoylarbutin
43	8.11	C_26_H_28_O_16_	595.4	301.1[M − H − Ara-Gal]^−^	Quercetin-3*-O-*arabinosylgalactoside
44	8.13	C_45_H_36_O_18_	863.3	573.1[M − H − C_15_H_14_O_6_]^−^, 451.1[M − H − C_15_H_12_O_6_ − C_7_H_8_O_2_]^−^, 289.2[M − H − C_15_H_12_O_6_ − C_15_H_10_O_6_]^−^	A-type procyanidin trimer
45	8.19	C_30_H_24_O_12_	575.3	448.9[M − H − C_6_H_6_O_3_]^−^, 285.1[M − H − C_15_H_14_O_6_]^−^	A-type procyanidin dimer
46	8.32	C_15_H_20_O_10_	359.3	197.2[M − H − hexose]^−^	Syringic acid*-O-*hexoside
47	8.41	C_9_H_8_O_3_	163.0	118.9[M − H − CO_2_]^−^	Hydroxycinnamic acid
48	8.43	C_17_H_20_O_9_	367.4	205.0[M − H − caffeoyl]^−^	3*-O-*Caffeoylquinic acid methyl ester
49	8.63	C_26_H_28_O_16_	595.4	301.1[M − H − Ara-Glc]^−^	Quercetin-3*-O-*arabinosylglucoside
50	8.65	C_27_H_30_O_16_	609.4	301.0[M − H − Rha-Gal]	Quercetin-3*-O-*rhamnosylgalactoside
51	8.72	C_21_H_20_O_11_	447.3	285.2[M − H − Glc]^−^	Luteolin-7*-O-*galactoside
52	8.87	C_27_H_30_O_16_	609.4	301.0[M − H − Rut]^−^	Rutin ^a^
53	8.91	C_45_H_38_O_18_	865.3	577.1[M − H − C_15_H_12_O_6_]^−^, 451.1[M − H − C_15_H_12_O_6_ − C_6_H_6_O_3_]^−^, 407.1[M − H − C_15_H_12_O_6_ − C_8_H_8_O_3_ − H_2_O]^−^, 289.0[M − H − 2C_15_H_12_O_6_]^−^	B-type procyanidin trimer
54	8.93	C_25_H_24_O_11_	499.4	353.1[M − H − *p*-coumaroyl]^−^, 337.2[M − H − caffeoyl]^−^, 191.2[M − H − caffeoyl − *p*-coumaroyl]^−^, 163.1[*p*-coumaroyl − H]^−^	*p*-Coumaroylcaffeoylquinic acid
55	8.98	C_21_H_20_O_12_	463.2	301.2[M − H − Gal]^−^	Quercetin-3*-O-*galactoside (Hyperoside) ^a^
56	9.01	C_27_H_30_O_15_	593.3	285.1[M − H − Rha-Gal]^−^	Kaempferol-3-*O*-rhamnosylgalactoside
57	9.14	C_21_H_22_O_10_	433.3	323.4[M − H − C_6_H_6_O_2_]^−^, 161.2[M − H − arbutin − H_2_O]^−^, 133.2[M − H − arbutin − H_2_O − CO]^−^	Caffeoylarbutin
58	9.15	C_25_H_24_O_11_	499.4	353. 1[M − H − *p*-coumaroyl]^−^, 336.9[M − H − caffeoyl]^−^, 191.1[M − H − caffeoyl − *p*-coumaroyl]^−^, 163.0[*p-*coumaroyl − H]^−^	*p*-Coumaroylcaffeoylquinic acid
59	9.16	C_21_H_20_O_12_	463.2	301.2[M − H − Glc]^−^	Quercetin-3*-O-*glucoside
60	9.17	C_30_H_26_O_12_	577.3	425.1[M − H − C_8_H_8_O_3_]^−^, 289.0[M − H − C_15_H_12_O_6_]^−^	B-type procyanidin dimer
61	9.25	C_25_H_24_O_12_	515.5	353.1[M − H − caffeoyl]^−^, 191.0[M − H − 2caffeoyl]^−^	Di*-O-*caffeoylquinic acid
62	9.26	C_21_H_20_O_11_	447.3	285.0[M − H − Glc]^−^	Luteolin-7*-O-*glucoside (Luteoloside) ^a^
63	9.30	C_45_H_38_O_18_	865.3	577.2[M − H − C_15_H_12_O_6_]^−^, 406.9[M − H − C_15_H_12_O_6_ − C_8_H_8_O_3_ − H_2_O]^−^, 289.0[M − H − 2C_15_H_12_O_6_]^−^	B-type procyanidin trimer
64	9.38	C_27_H_30_O_15_	593.3	285.1[M − H − Rha-Glc]^−^	Kaempferol-3-*O*-rhamnosylglucoside
65	9.44	C_21_H_18_O_11_	445.3	401.0[M − H − CO_2_]^−^, 357.2[M − H − 2CO_2_]^−^, 313.2[M − H − 3CO_2_]^−^, 225.2[M − H − C_6_H_8_O_6_ − CO_2_]^−^, 181.1[M − H − C_6_H_8_O_6_ − 2CO_2_]^−^	Apigenin*-O-*glucuronide or isomer
66	9.46	C_30_H_24_O_12_	575.3	539.0[M − H − H_2_O − H_2_O]^−^, 449.1[M − H − C_6_H_6_O_3_]^−^, 407.1[M − H − CO_2_ − C_7_H_8_O_2_]^−^, 285.1[M − H − C_15_H_14_O_6_]^−^	A-type procyanidin dimer
67	9.51	C_45_H_34_O_18_	861.3	735.1[M − H − C_6_H_6_O_3_]^−^, 693.2[M − H − CO_2_ − C_7_H_8_O_2_]^−^, 571.2[M − H − C_15_H_14_O_6_]^−^	A-type procyanidin trimer
68	9.54	C_21_H_22_O_9_	417.2	307.3[M − H − C_6_H_6_O_2_]^−^, 163.1[M − H − arbutin]^−^, 145.1[M − H − arbutin − H_2_O]^−^	*p*-Coumaroylarbutin
69	9.65	C_17_H_20_O_9_	367.4	205.0[M − H − caffeoyl]^−^, 191.1[M − H − caffeoyl − methyl]^−^	5*-O-*Caffeoylquinic acid methyl ester
70	9.69	C_45_H_36_O_18_	863.3	575.0[M − H − C_15_H_12_O_6_]^−^, 449.0[M − H − C_15_H_12_O_6_ − C_6_H_6_O_3_]^−^	A-type procyanidin trimer
71	9.70	C_19_H_16_O_12_	435.3	273.1[M − H − caffeoyl]^−^, 205.3[M − H − caffeoylmalonyl]^−^, 161.1[M − H − caffeoylmalonyl − CO_2_]^−^	Caffeoyl-malonyl-methylcitric acid
72	9.75	C_21_H_18_O_11_	445.3	401.2[M − H − CO_2_]^−^, 357.2[M − H − 2CO_2_]^−^, 313.1[M − H − 3CO_2_]^−^, 225.1[M − H − C_6_H_8_O_6_ − CO_2_]^−^	Apigenin*-O-*glucuronide or isomer
73	9.76	C_27_H_30_O_15_	593.3	285.1[M − H − Rut]^−^	Kaempferol-3-*O*-rutinoside ^a^
74	9.78	C_23_H_22_O_13_	505.3	445.2[M − H − CH_3_COOH]^−^, 301.1[M − H − acetyl-Gal]^−^	Quercetin-acylated-galactoside
75	9.80	C_28_H_32_O_16_	623.5	315.0[M − H − Gal-Rha]^−^	Isorhamnetin-3*-O-*rhamnosylgalactoside
76	9.87	C_25_H_24_O_12_	515.5	353.0[M − H − caffeoyl]^−^	3,4*-O-*Dicaffeoylquinic acid (Isochlorogenic acid B) ^a^
77	9.92	C_27_H_30_O_14_	577.3	269.1[M − H − Rut]^−^	Apigenin rutinoside
78	9.95	C_28_H_32_O_16_	623.5	315.0[M − H − Rut]^−^	Isorhamnetin-3*-O-*rutinoside ^a^
79	10.07	C_21_H_20_O_11_	447.3	285.0[M − H − Glc]^−^	Kaempferol-3*-O-*galactoside
80	10.09	C_23_H_22_O_13_	505.3	445.1[M − H − CH_3_COOH]^−^, 301.1[M − H − acetyl-Glc]^−^	Quercetin-acylated-glucoside
81	10.10	C_22_H_22_O_12_	477.3	315.0[M − H − Gal]^−^	Isorhamnetin-3*-O-*galactoside
82	10.14	C_28_H_32_O_15_	607.4	299.1[M − H − Nhe]^−^	Chrysoeriol-7-neohesperidoside
83	10.21	C_25_H_24_O_12_	515.5	353.2[M − H − caffeoyl]^−^, 191.0[M − H − 2caffeoyl]^−^, 178.9[M − H − caffeoyl-quinoyl]^−^	3,5*-O-*Dicaffeoylquinic acid (Isochlorogenic acid A) ^a^
84	10.22	C_30_H_24_O_12_	575.3	406.9[M − H − CO_2_ − C_7_H_8_O_2_]^−^	A-type procyanidin dimer
85	10.27	C_21_H_22_O_9_	417.2	307.3[M − H − C_6_H_6_O_2_]^−^, 163.0[M − H − arbutin]^−^, 145.1[M − H − arbutin − H_2_O]^−^	*p*-Coumaroylarbutin
86	10.28	C_22_H_22_O_12_	477.3	315.0[M − H − Glc]^−^	Isorhamnetin-3*-O-*glucoside
87	10.31	C_21_H_20_O_11_	447.3	285.0[M − H − hexose]^−^	Kaempferol-3*-O-*glucoside
88	10.34	C_21_H_20_O_10_	431.2	269.1[M − H − hexose]^−^	Apigenin*-O-*hexoside
89	10.38	C_25_H_24_O_11_	499.4	353. 0[M − H − *p*-coumaroyl]^−^, 337.0[M − H − caffeoyl]^−^, 191.0[M − H − caffeoyl − *p*-coumaroyl]^−^	*p*-Coumaroylcaffeoylquinic acid
90	10.58	C_22_H_22_O_11_	461.4	298.9[M − H − Gal]^−^	Chrysoeriol-7*-O-*galactoside
91	10.60	C_45_H_34_O_18_	861.3	735.0[M − H − C_6_H_6_O_3_]^−^, 693.1[M − H − CO_2_ − C_7_H_8_O_2_]^−^, 571.1[M − H − C_15_H_14_O_6_]^−^, 288.9[M − H − 2C_15_H_10_O_6_]^−^	A-type procyanidin trimer
92	10.71	C_25_H_24_O_12_	515.5	353.1[M − H − caffeoyl]^−^, 191.1[M − H − 2caffeoyl]^−^, 179.0[M − H − caffeoyl-quinoyl]^−^	4,5*-O-*Dicaffeoylquinic acid (Isochlorogenic acid C) ^a^
93	10.75	C_22_H_22_O_11_	461.4	299.2[M − H − Glc]^−^	Chrysoeriol-7*-O-*glucoside
94	10.80	C_23_H_22_O_12_	489.1	285.1[M − H − acetyl-Gal]^−^	Kaempferol-acylated-galactoside
95	10.85	C_24_H_24_O_13_	519.4	315.3[M − H − acetyl-Gal]^−^, 299.2[M − H − acetyl-Gal − CH_4_]^−^	Isorhamnetin-acylated-galactoside
96	11.05	C_24_H_24_O_13_	519.5	315.0[M − H − acetyl-Glc]^−^, 299.2[M − H − acetyl-Glc − CH_4_]^−^, 271.2[M − H − acetyl-Glc − CH_4_ − H_2_O]^−^	Isorhamnetin-acylated-glucoside
97	11.30	C_25_H_24_O_11_	499.4	353. 0[M − H − *p*-coumaroyl]^−^, 337.1[M − H − caffeoyl]^−^, 190.9[M − H − caffeoyl − *p*-coumaroyl]^−^, 163.0[*p−*coumaroyl − H]^−^	*p*-Coumaroylcaffeoylquinic acid
98	11.78	C_23_H_22_O_12_	489.1	285.1[M − H − acetyl-Glc]^−^	Kaempferol-acylated-glucoside
99	11.80	C_17_H_20_O_9_	367.4	205.1[M − H − caffeoyl]^−^, 190.9[M − H − caffeoyl-methyl]^−^, 147.0[M − H − caffeoyl-methyl − CO_2_]^−^	1-*O-*Caffeoylquinic acid methyl ester
100	12.13	C_21_H_20_O_11_	447.3	295.4[M − H − Galloyl]^−^	Galloyl-coumaric acid pentoside
101	12.15	C_22_H_22_O_12_	477.3	273.1[M − H − acetyl-hexose]^−^	Phloretin-acylated-hexoside
102	12.38	C_21_H_20_O_11_	447.3	295.1[M − H − Galloyl]^−^	Galloyl-coumaric acid pentoside
***Triterpenoid***					
1′	1.21	C_30_H_48_O_5_	487.3	469.1[M − H − H_2_O]^−^, 425.0[M − H − H_2_O − CO_2_]^−^	Euscaphic acid
2′	1.40	C_30_H_48_O_5_	487.3	469.3[M − H − H_2_O]^−^, 425.2[M − H − H_2_O − CO_2_]^−^	Tormentic acid
3′	1.59	C_30_H_46_O_5_	485.3	467.1[M − H − H_2_O]^−^, 423.2[M − H − H_2_O − CO_2_]^−^	Anmurcoic acid
4′	1.95	C_30_H_48_O_4_	471.3	453.2[M − H − H_2_O]^−^, 407.0[M − H − H_2_O − CH_2_O]^−^	Pomolic acid isomer
5′	2.47	C_30_H_46_O_4_	469.3	425.1[M − H − CO2]^−^, 407.0[M − H − H_2_O − CO_2_]^−^	1-Hydroxy-3-oxours-12-en-28-oic acid or isomer
6′	2.63	C_30_H_48_O_4_	471.3	453.3[M − H − H_2_O]^−^, 407.1[M − H − H_2_O − CH_2_O]^−^	Pomolic acid ^a^
7′	2.89	-	701.5	641.2, 555.1, 540.1, 481.1	Unknown
8′	3.11	C_30_H_48_O_4_	471.3	453.2[M − H − H_2_O]^−^, 407.1[M − H − H_2_O − CH_2_O]^−^	Alphitolic acid ^a^
9′	3.45	C_30_H_48_O_4_	471.3	453.0[M − H − H_2_O]^−^, 407.1[M − H − H_2_O − CH_2_O]^−^	Maslinic acid ^a^
10′	3.79	C_30_H_48_O_4_	471.3	453.2[M − H − H_2_O]^−^, 407.3[M − H − H_2_O − CH_2_O]^−^	Corosolic acid
11′	4.38	C_30_H_46_O_4_	469.3	425.2[M − H − CO_2_]^−^, 407.1[M − H − CO_2_ − H_2_O]^−^	1-Hydroxy-3-oxours-12-en-28-oic acid or isomer
12′	4.79	C_30_H_46_O_4_	469.3	425.1[M − H − CO_2_]^−^, 407.3[M − H − CO_2_ − H_2_O]^−^	1-Hydroxy-3-oxours-12-en-28-oic acid or isomer
13′	6.89	C_30_H_48_O_3_	455.3	407.4[M − H − H_2_O − CH_2_O]^−^	Betulinic acid ^a^
14′	7.44	C_30_H_48_O_3_	455.3	407.4[M − H − H_2_O − CH_2_O]^−^	Oleanolic acid ^a^
15′	7.95	C_30_H_48_O_3_	455.3	407.2[M − H − H_2_O − CH_2_O]^−^	Ursolic acid ^a^
16′	8.37	-	687.4	627.1, 541.2, 526.2, 467.0	Unknown

Note: ^#^ the proposal ion was [M − H + HCOOH]^−^; ^a^ identification confirmed by authentic standards.

**Table 2:** Calibration curves, LODs and LOQs data used for UPLC-MS/MS quantification of polyphenols and triterpenoids.

Analyte	Ion Transition	Calibration Curve	*R* ^2^	Linear Range (ng/mL)	LOD (ng/mL)	LOQ (ng/mL)
Quinic acid	191 > 93	y = 10.2 x + 0.06	0.9996	7843.1–784,313.6	13.1	78.4
Arbutin	317 > 161	y = 19.9 x + 0.6	0.9999	3919.2–391,921.6	16.3	98.0
(+)-Catechin	289 > 289	y = 270.9 x − 0.7	0.9997	73.5–14,703.9	2.5	7.4
(−)-Epicatechin	289 > 289	y = 246.8 x + 1.7	0.9998	4.9–49,166.6	1.6	4.9
Chlorogenic acid	353 > 179	y = 1.3 x − 1.1	0.9996	3923.1–196,156.8	65.4	392.3
Cryptochlorogenic acid	353 > 179	y = 101.1 x + 30.0	0.9998	32.6–1302.6	10.9	32.6
Neochlorogenic acid	353 > 173	y = 128.7 x + 4.1	0.9999	25.7–1029.8	8.6	25.7
Isochlorogenic acid A	515 > 353	y = 82.1 x − 0.9	0.9998	66.9–33,427.8	5.6	33.4
Isochlorogenic acid B	515 > 353	y = 287.7 x + 69.3	0.9998	10.5–524.3	3.5	10.5
Isochlorogenic acid C	515 > 353	y = 267.2 x + 43.2	0.9997	17.3–690.3	2.9	17.3
*p*-Coumaric acid	163 > 163	y = 1270.4 x + 90.3	0.9998	2.0–9817.6	0.2	2.0
Caffeic acid	179 > 179	y = 165.7 x + 15.4	0.9998	9.8–98,352.8	1.6	9.8
Rutin	609 > 300	y = 365.1 x + 2.1	0.9999	19.7–4912.7	1.6	9.8
	609 > 609	y = 478.3 x + 53.1	0.9998	4.9–9825.5	1.6	4.9
Kaempferol-3-*O*-rutinoside	593 > 285	y = 36.2 x + 0.7	0.9997	49.4–4941.2	19.8	49.4
	593 > 593	y = 90.2 x + 2.3	0.9997	19.8–4941.2	9.9	19.8
Isorhamnetin-3-*O*-rutinoside	623 > 315	y = 136.3 x + 5.3	0.9998	20.0–9982.3	5.0	20.0
	623 > 623	y = 342.7 x − 12.9	0.9999	10.0–9982.3	5.0	10.0
Luteoloside	447 > 285	y = 646.2 x + 95.2	0.9997	5.0–981.2	1.6	4.9
	447 > 447	y = 504.6 x − 11.0	0.9998	5.0–981.2	1.6	4.9
Hyperoside	463 > 300	y = 518.6 x + 20.7	0.9995	9.8–978.6	3.3	9.8
Oleanic acid	455 > 455	y = 2546.2 x − 63.3	0.9999	9.8–982.2	0.8	2.0
Ursolic acid	455 > 455	y = 2329.5 x + 44.9	0.9999	0.8–987.6	0.3	0.8
Betulinic acid	455 > 455	y = 4256.9 x − 540.1	1.0000	0.8–489.7	0.3	0.8
Pomolic acid	471 > 471	y = 1302.8 x − 22.8	0.9999	2.0–198.3	0.8	2.0
Maslinic acid	471 > 471	y = 1255.5 x − 2.9	0.9999	2.0–493.1	0.8	2.0
Corosolic acid	471 > 471	y = 1196.7 x − 8.6	0.9999	2.0–493.6	0.8	2.0

**Table 3:** The content of chemical constituents in thinned young pears of ten different varieties (mean ± SD, *n* = 3).

Peak NO.	Compound	The Compound Content in Different Pear Varieties (μg/g FW)										
		DSSL	YL	CG	FS	NGL	XSL	KELXL	XJSL	HQL	SJL	Mean
***Phenolic acid***												
1	Quinic acid	8798.1 ± 122.4	9532.7 ± 74.3	8788.1 ± 74.6	8892.3 ± 85.8	10,500.5 ± 93.4	8984.8 ± 68.8	6725.8 ± 44.4	6064.2 ± 49.4	4991.7 ± 49.5	8666.34 ± 103.37	8194.5
2	Cinnamic acid isomer	120.0 ± 1.7	153.2 ± 2.6	130.7 ± 1.9	144.4 ± 2.1	60.4 ± 0.6	90.4 ± 0.8	56.3 ± 0.8	30.9 ± 0.6	217.4 ± 4.2	203.8 ± 1.9	120.8
5	Cryptochlorogenic acid	5.3 ± 0.1	10.5 ± 0.2	5.7 ± 0.1	4.0 ± 0.1	2.6 ± 0.1	4.5 ± 0.1	6.7 ± 0.1	6.8 ± 0.1	8.3 ± 0.2	6.3 ± 0.2	6.1
10	Caffeoylglycerol	4.6 ± 0.1	N.Q.	19.9 ± 0.4	14.4 ± 0.2	N.Q.	5.4 ± 0.1	9.12 ± 0.1	4.2 ± 0.1	25.6 ± 0.2	31.2 ± 0.3	11.5
13	Chlorogenic acid	3988.5 ± 33.9	3816.4 ± 50.6	1783.6 ± 16.2	1162.7 ± 9.1	4139.0 ± 39.9	3760.7 ± 28.2	1207.7 ± 14.3	2311.6 ± 17.1	2564.1 ± 28.1	2485.3 ± 23.5	2722.0
15	Neochlorogenic acid	2.8 ± 0.1	3.4 ± 0.1	1.6 ± 0.1	1.0 ± 0.0	2.1 ± 0.0	3.2 ± 0.1	1.7 ± 0.0	2.8 ± 0.0	1.8 ± 0.1	2.0 ± 0.0	2.2
16	*p*-Coumaroylcaffeoyl malate	N.D.	N.D.	N.D.	N.D.	N.D.	N.D.	N.Q.	5.6 ± 0.1	10.8 ± 0.1	8.7 ± 0.2	2.5
20	1*-O-*Caffeoylquinic acid	26.2 ± 0.3	22.4 ± 0.3	12.2 ± 0.2	14.8 ± 0.2	20.5 ± 0.2	28.2 ± 0.3	10.9 ± 0.1	24.0 ± 0.3	23.2 ± 0.2	24.3 ± 0.2	20.7
22	Caffeoylshikimic acid	1.0 ± 0.0	N.Q.	0.9 ± 0.0	1.0 ± 0.0	0.7 ± 0.0	0.7 ± 0.0	2.0 ± 0.0	0.8 ± 0.0	1.8 ± 0.1	1.4 ± 0.0	1.0
25	4-*p*-Coumaroylquinic acid	28.1 ± 0.2	32.4 ± 0.4	5.2 ± 0.1	3.0 ± 0.1	22.5 ± 0.5	17.2 ± 0.2	4.8 ± 0.1	8.6 ± 0.1	34.3 ± 0.5	22.4 ± 0.2	17.8
28	Caffeoylshikimic acid	25.7 ± 0.2	19.0 ± 0.2	32.8 ± 0.5	20.4 ± 0.2	13.5 ± 0.2	27.6 ± 0.3	7.4 ± 0.2	5.0 ± 0.1	8.3 ± 0.1	12.5 ± 0.1	17.2
31	3*-O-*Feruloylquinic acid	49.9 ± 0.6	41.2 ± 0.5	12.8 ± 0.2	5.0 ± 0.1	49.6 ± 0.8	51.8 ± 0.9	6.5 ± 0.1	9.8 ± 0.2	19.9 ± 0.3	53.2 ± 0.9	30.0
32	Caffeoyl-malonyl-methylcitric acid	4.9 ± 0.1	5.3 ± 0.1	1.7 ± 0.0	0.5 ± 0.0	7.2 ± 0.1	6.4 ± 0.1	0.7 ± 0.0	1.2 ± 0.0	2.3 ± 0.0	6.3 ± 0.1	3.7
34	5-*p*-Coumaroylquinic acid	1.0 ± 0.0	1.1 ± 0.0	0.4 ± 0.0	0.5 ± 0.0	0.8 ± 0.0	0.6 ± 0.0	0.5 ± 0.0	0.6 ± 0.0	3.1 ± 0.0	2.3 ± 0.1	1.1
36	4*-O-*Caffeoylquinic acid methyl ester	1.9 ± 0.1	0.9 ± 0.0	0.9 ± 0.0	1.4 ± 0.0	2.4 ± 0.0	2.3 ± 0.0	1.4 ± 0.1	1.4 ± 0.0	6.4 ± 0.2	7.7 ± 0.2	2.7
47	Hydroxycinnamic acid	1.1 ± 0.0	N.D.	1.4 ± 0.0	1.7 ± 0.0	1.2 ± 0.0	1.5 ± 0.0	0.6 ± 0.0	5.7 ± 0.1	3.4 ± 0.0	1.9 ± 0.0	1.8
48	3*-O-*Caffeoylquinic acid methyl ester	N.D.	N.D.	N.D.	N.D.	N.D.	N.D.	N.D.	N.Q.	5.0 ± 0.1	5.1 ± 0.1	1.0
54	*p*-Coumaroylcaffeoylquinic acid	N.D.	N.D.	N.D.	N.D.	1.3 ± 0.0	N.Q.	0.6 ± 0.0	N.D.	N.D.	N.D.	0.2
58	*p*-Coumaroylcaffeoylquinic acid	N.D.	N.D.	N.D.	N.D.	4.5 ± 0.1	N.Q.	2.1 ± 0.0	N.Q.	N.D.	N.D.	0.7
61	Di*-O-*caffeoylquinic acid	3.1 ± 0.1	N.D.	N.D.	N.D.	1.7 ± 0.0	N.Q.	3.0 ± 0.0	N.Q.	N.D.	N.D.	0.8
69	5*-O-*Caffeoylquinic acid methyl ester	6.2 ± 0.2	N.Q.	1.7 ± 0.0	9.6 ± 0.2	44.1 ± 0.7	N.Q.	12.6 ± 0.2	N.Q.	17.5 ± 0.2	13.3 ± 0.2	10.5
71	Caffeoyl-malonyl-methylcitric acid	11.6 ± 0.2	N.Q.	3.9 ± 0.0	18.6 ± 0.2	87.7 ± 0.9	1.3 ± 0.0	23.1 ± 0.2	N.Q.	34.7 ± 0.3	26.2 ± 0.3	20.7
76	Isochlorogenic acid B	0.7 ± 0.0	0.6 ± 0.0	0.4 ± 0.0	0.8 ± 0.0	N.D.	N.Q.	0.5 ± 0.0	N.Q.	0.5 ± 0.0	N.Q.	0.4
83	Isochlorogenic acid A	139.2 ± 2.2	240.8 ± 2.6	52.9 ± 1.3	34.8 ± 0.3	21.1 ± 0.3	60.4 ± 0.7	74.3 ± 0.8	52.8 ± 0.5	116.0 ± 1.4	89.2 ± 2.0	88.2
89	*p*-Coumaroylcaffeoylquinic acid	0.8 ± 0.0	0.6 ± 0.0	N.D.	N.D.	1.5 ± 0.0	N.Q.	N.Q.	N.Q.	1.1 ± 0.1	0.6 ± 0.0	0.5
92	Isochlorogenic acid C	3.2 ± 0.1	4.2 ± 0.1	2.8 ± 0.0	1.8 ± 0.1	2.0 ± 0.0	1.6 ± 0.0	2.4 ± 0.1	1.6 ± 0.0	1.6 ± 0.0	1.3 ± 0.0	2.3
97	*p*-Coumaroylcaffeoylquinic acid	17.2 ± 0.2	21.3 ± 0.2	4.8 ± 0.1	3.1 ± 0.1	2.3 ± 0.0	3.9 ± 0.1	8.1 ± 0.1	3.8 ± 0.0	18.2 ± 0.2	11.2 ± 0.2	9.4
99	1*-O-*Caffeoylquinic acid methyl ester	1.2 ± 0.0	N.Q.	N.Q.	1.6 ± 0.0	5.4 ± 0.1	N.Q.	3.4 ± 0.1	N.Q.	6.8 ± 0.2	5.4 ± 0.1	2.4
Total phenolic acids		13,242.2 ± 117.8	13,905.9 ± 96.8	10,864.4 ± 86.4	10,337.2 ± 95.9	14,994.6 ± 99.2	13,052.6 ± 72.1	8172.6 ± 52.8	8541.3 ± 61.4	8123.9 ± 50.4	11,688.1 ± 89.6	11,292.3
***Phenolic glycoside***												
3	Arbutin	3925.2 ± 70.1	5605.5 ± 53.9	4843.8 ± 80.4	4945.5 ± 78.1	5899.1 ± 71.3	5905.9 ± 60.1	3007.4 ± 49.4	2285.6 ± 22.3	3511.6 ± 57.3	4510.3 ± 31.6	4444.0
6	Dihydro-caffeoyl*-O-*hexoside	9.1 ± 0.2	47.6 ± 0.5	25.9 ± 0.2	3.8 ± 0.1	48.4 ± 0.6	1.9 ± 0.1	1.4 ± 0.0	3.3 ± 0.1	19.2 ± 0.4	12.2 ± 0.1	17.3
8	Hydroxyphenylpropionic acid*-O-*hexoside	2.5 ± 0.0	0.4 ± 0.0	0.4 ± 0.0	0.3 ± 0.0	1.3 ± 0.1	0.5 ± 0.0	0.2 ± 0.0	0.2 ± 0.0	0.3 ± 0.0	0.4 ± 0.0	0.6
9	Syringic acid*-O-*hexoside	3.8 ± 0.1	N.Q.	17.7 ± 0.2	12.7 ± 0.3	N.Q.	3.5 ± 0.1	7.6 ± 0.2	3.4 ± 0.1	21.7 ± 0.6	27.7 ± 0.5	9.8
11	Dihydro-caffeoyl*-O-*hexoside	8.8 ± 0.1	7.9 ± 0.1	4.9 ± 0.1	4.2 ± 0.1	8.7 ± 0.1	3.6 ± 0.1	10.1 ± 0.1	4.6 ± 0.1	2.8 ± 0.1	5.2 ± 0.1	6.1
21	Caffeoyl*-O-*hexoside	8.9 ± 0.1	12.9 ± 0.1	4.8 ± 0.1	4.8 ± 0.1	5.1 ± 0.1	5.6 ± 0.1	2.2 ± 0.0	N.Q.	3.8 ± 0.1	5.0 ± 0.1	5.3
23	Roseoside	0.3 ± 0.0	0.7 ± 0.0	0.4 ± 0.0	0.3 ± 0.0	0.4 ± 0.0	0.4 ± 0.0	0.4 ± 0.0	0.3 ± 0.0	0.9 ± 0.0	1.3 ± 0.0	0.5
26	Syringic acid*-O-*hexoside	1.4 ± 0.0	1.6 ± 0.0	0.3 ± 0.0	0.2 ± 0.0	1.1 ± 0.0	0.8 ± 0.0	0.3 ± 0.0	0.5 ± 0.0	1.8 ± 0.1	1.0 ± 0.0	0.9
39	Caffeoylarbutin	N.D.	N.D.	N.D.	N.D.	N.Q.	N.D.	N.D.	7.0 ± 0.1	N.D.	44.0 ± 0.5	5.1
42	Caffeoylarbutin	193.7 ± 2.7	N.Q.	201.1 ± 1.5	239.1 ± 2.5	N.Q.	74.1 ± 0.7	225.7 ± 1.7	126.9 ± 2.1	N.Q.	2.6 ± 0.1	106.3
46	Syringic acid*-O-*hexoside	0.4 ± 0.0	0.7 ± 0.0	0.6 ± 0.0	0.5 ± 0.0	0.7 ± 0.0	0.6 ± 0.0	0.2 ± 0.0	N.Q.	N.Q.	0.2 ± 0.0	0.4
57	Caffeoylarbutin	9.9 ± 0.1	N.Q.	13.9 ± 0.2	14.9 ± 0.4	N.Q.	5.2 ± 0.1	13.5 ± 0.2	8.9 ± 0.2	N.Q.	2.8 ± 0.1	6.9
68	*p*-Coumaroylarbutin	33.9 ± 0.4	0.4 ± 0.0	34.8 ± 0.5	50.9 ± 0.8	1.0 ± 0.0	12.1 ± 0.2	39.7 ± 0.7	20.0 ± 0.2	0.4 ± 0.0	0.3 ± 0.0	19.3
85	*p*-Coumaroylarbutin	11.1 ± 0.2	N.Q.	14.0 ± 0.2	18.7 ± 0.4	0.3 ± 0.0	7.0 ± 0.1	17.4 ± 0.2	9.8 ± 0.1	1.3 ± 0.0	2.1 ± 0.1	8.2
100	Galloyl-coumaric acid pentoside	0.3 ± 0.0	0.3 ± 0.0	0.2 ± 0.0	0.1 ± 0.0	0.2 ± 0.0	0.1 ± 0.0	0.1 ± 0.0	0.4 ± 0.0	0.3 ± 0.0	0.6± 0.0	0.3
102	Galloyl-coumaric acid pentoside	0.1 ± 0.0	0.1 ± 0.00	0.1 ± 0.0	N.Q.	0.2 ± 0.0	0.1 ± 0.0	0.1 ± 0.0	0.3 ± 0.0	0.3 ± 0.0	0.3 ± 0.0	0.2
Total phenolic glycosides		4209.2 ± 72.9	5678.1 ± 53.6	5162.8 ± 83.5	5296.0 ± 77.7	5966.6 ± 71.3	6021.3 ± 59.5	3326.3 ± 50.8	2471.0 ± 23.4	3564.4 ± 56.8	4616.0 ± 32.1	4631.2
***Flavone***												
38	Quercetin-3*-O-*xylosylrhamnosylglucoside	1.7 ± 0.0	1.4 ± 0.0	2.6 ± 0.1	1.5 ± 0.0	1.1 ± 0.0	2.7 ± 0.0	4.8 ± 0.1	N.Q.	N.D.	N.D.	1.6
41	Isorhamnetin-acylated-hexoside	1.1 ± 0.0	N.Q.	1.1 ± 0.1	1.3 ± 0.0	N.Q.	0.4 ± 0.0	1.2 ± 0.0	0.7 ± 0.0	N.D.	N.D.	0.6
43	Quercetin-3*-O-*arabinosylgalactoside	5.9 ± 0.1	2.0 ± 0.1	5.1 ± 0.1	2.6 ± 0.1	1.0 ± 0.0	2.7 ± 0.1	3.4 ± 0.1	N.Q.	N.D.	N.D.	2.3
49	Quercetin-3*-O-*arabinosylglucoside	N.D.	N.D.	N.D.	N.D.	N.D.	N.D.	N.D.	N.D.	0.4 ± 0.0	0.3 ± 0.0	0.1
50	Quercetin-3*-O-*rhamnosylgalactoside	0.3 ± 0.0	0.5 ± 0.0	1.0 ± 0.0	0.3 ± 0.0	0.4 ± 0.0	2.2 ± 0.1	0.5 ± 0.0	N.Q.	16.9 ± 0.5	6.9 ± 0.1	2.9
51	Luteolin-7*-O-*galactoside	N.D.	N.D.	N.D.	N.D.	0.8 ± 0.0	N.Q.	N.D.	N.D.	N.D.	N.D.	0.1
52	Rutin	12.6 ± 0.1	10.1 ± 0.2	13.3 ± 0.2	8.3 ± 0.2	12.3 ± 0.2	19.7 ± 0.2	24.5 ± 0.4	1.6 ± 0.0	61.9 ± 1.1	61.2 ± 0.3	22.5
55	Hyperoside	2.7 ± 0.0	1.4 ± 0.0	2.0 ± 0.1	1.0 ± 0.0	0.4 ± 0.0	1.5 ± 0.0	0.4 ± 0.0	3.0 ± 0.1	11.4 ± 0.2	8.8 ± 0.1	3.2
56	Kaempferol-3*-O-*rhamnosylgalactoside	63.9 ± 1.7	N.Q.	N.D.	N.D.	12.1 ± 0.1	N.Q.	17.7 ± 0.3	N.Q.	N.Q.	N.Q.	9.4
59	Quercetin-3*-O-*glucoside	20.6 ± 0.2	7.9 ± 0.2	16.5 ± 0.3	7.7 ± 0.2	5.3 ± 0.1	12.3 ± 0.2	6.5 ± 0.1	16.1 ± 0.3	56.1 ± 0.7	50.4 ± 0.5	19.9
62	Luteoloside	14.1 ± 0.1	N.Q.	N.D.	N.D.	5.8 ± 0.1	N.Q.	11.5 ± 0.1	N.Q.	N.D.	N.D.	3.2
64	Kaempferol-3*-O-r*hamnosylglucoside	N.D.	N.D.	N.D.	N.D.	N.D.	N.D.	N.D.	N.D.	1.95 ± 0.1	1.3 ± 0.1	0.3
65	Apigenin*-O-*glucuronide or isomer	N.D.	N.D.	N.D.	N.D.	0.2 ± 0.0	N.D.	N.D.	N.D.	0.4 ± 0.0	0.3 ± 0.0	0.1
72	Apigenin*-O-*glucuronide or isomer	0.5 ± 0.0	0.3 ± 0.0	0.3 ± 0.0	0.2 ± 0.0	N.D.	0.5 ± 0.0	N.D.	N.D.	3.3 ± 0.1	1.5 ± 0.0	0.7
73	Kaempferol-3*-O-*rutinoside	2.3 ± 0.1	1.7 ± 0.1	2.8 ± 0.1	2.7 ± 0.1	2.8 ± 0.1	1.2 ± 0.0	4.5 ± 0.2	4.0 ± 0.1	17.4 ± 0.5	15.9 ± 0.4	5.5
74	Quercetin-acylated-galactoside	5.98 ± 0.1	2.1 ± 0.0	3.3 ± 0.0	1.6 ± 0.0	0.9 ± 0.0	4.8 ± 0.1	N.D.	N.D.	39.4 ± 0.9	16.1 ± 0.2	7.4
75	Isorhamnetin-3*-O-*rhamnosylgalactoside	10.8 ± 0.2	21.3 ± 0.4	10.2 ± 0.2	7.3 ± 0.1	8.7 ± 0.1	2.3 ± 0.1	12.6 ± 0.4	15.8 ± 0.2	20.2 ± 0.2	58.8 ± 0.7	16.8
77	Apigenin rutinoside	3.8 ± 0.1	N.Q.	N.D.	N.D.	1.2 ± 0.0	N.Q.	N.D.	N.D.	N.Q.	1.0 ± 0.0	0.6
78	Isorhamnetin-3*-O-*rutinoside	86.9 ± 1.2	98.1 ± 1.1	49.9 ± 0.7	66.4 ± 1.0	75.3 ± 1.7	9.1 ± 0.1	136.6 ± 1.6	62.9 ± 1.4	28.5 ± 0.5	143.8 ± 1.3	75.8
79	Kaempferol-3*-O-*galactoside	4.3 ± 0.1	1.7 ± 0.0	8.2 ± 0.1	8.2 ± 0.2	1.6 ± 0.1	3.5 ± 0.1	2.1 ± 0.1	12.8 ± 0.3	16.8 ± 0.5	8.1 ± 0.2	6.7
80	Quercetin-acylated-glucoside	0.5 ± 0.0	0.2 ± 0.0	0.3 ± 0.0	0.3 ± 0.0	0.3 ± 0.0	0.6 ± 0.0	N.Q.	N.Q.	3.1 ± 0.0	2.1 ± 0.1	0.8
81	Isorhamnetin-3*-O-*galactoside	5.7 ± 0.1	2.6 ± 0.1	2.6 ± 0.1	1.7 ± 0.0	0.6 ± 0.1	0.9 ± 0.1	0.4 ± 0.0	19.8 ± 0.2	1.4 ± 0.0	4.6 ± 0.1	4.0
82	Chrysoeriol-7-neohesperidoside	9.3 ± 0.2	N.Q.	N.Q.	N.Q.	2.7 ± 0.1	N.Q.	2.0 ± 0.0	N.Q.	N.Q.	N.Q.	1.4
86	Isorhamnetin-3*-O-*glucoside	21.3 ± 0.4	8.8 ± 0.2	13.0 ± 0.3	9.2 ± 0.2	4.6 ± 0.1	2.4 ± 0.0	3.7 ± 0.1	95.1 ± 0.9	7.4 ± 0.2	22.1 ± 0.4	18.8
87	Kaempferol-3*-O-*glucoside	2.4 ± 0.1	N.Q.	N.D.	1.1 ± 0.1	55.1 ± 0.9	N.Q.	2.6 ± 0.1	N.Q.	N.Q.	N.Q.	6.1
88	Apigenin*-O-*hexoside	9.8 ± 0.2	N.Q.	N.D.	N.D.	19.6 ± 0.4	N.Q.	3.5 ± 0.1	N.Q.	0.6 ± 0.1	0.7 ± 0.0	3.4
90	Chrysoeriol-7*-O-*galactoside	15.7 ± 0.2	N.Q.	N.D.	N.D.	14.7 ± 0.2	N.Q.	19.9 ± 0.1	N.Q.	N.Q.	N.Q.	5.0
93	Chrysoeriol-7*-O-*glucoside	0.5 ± 0.0	N.D.	N.D.	N.D.	22.9 ± 0.6	N.Q.	N.D.	N.D.	0.2 ± 0.0	0.3 ± 0.0	2.4
94	Kaempferol-acylated-galactoside	14.5 ± 0.4	7.1 ± 0.2	6.3 ± 0.1	8.3 ± 0.2	4.0 ± 0.1	2.4 ± 0.1	N.Q.	2.0 ± 0.1	150.5 ± 2.1	28.0 ± 0.3	22.3
95	Isorhamnetin-acylated-galactoside	6.2 ± 0.1	2.95 ± 0.1	1.3 ± 0.0	1.1 ± 0.0	N.D.	N.D.	N.D.	N.D.	3.8 ± 0.1	3.3 ± 0.1	1.9
96	Isorhamnetin-acylated-glucoside	53.7 ± 1.1	26.1 ± 0.2	15.1 ± 0.2	14.6 ± 0.3	4.8 ± 0.1	2.0 ± 0.1	N.Q.	3.1 ± 0.0	36.5 ± 0.7	55.6 ± 0.9	21.2
98	Kaempferol-acylated-glucoside	2.8 ± 0.1	N.Q.	N.D.	N.D.	N.D.	N.D.	5.4 ± 0.1	1.3 ± 0.0	N.Q.	1.2 ± 0.1	1.1
101	Phloretin-acylated-hexoside	1.1 ± 0.0	N.Q.	N.D.	2.1 ± 0.1	N.D.	N.D.	3.3 ± 0.1	N.D.	N.D.	N.D.	0.6
Total flavones		380.9 ± 4.7	196.1 ± 0.3	154.7 ± 1.1	147.3 ± 1.5	259.0 ± 3.1	71.1 ± 1.4	266.9 ± 2.0	238.3 ± 3.6	478.1 ± 3.7	492.2 ± 1.6	268.5
***Flavan-3-ol***												
4	A-type procyanidin dimer	N.D.	N.D.	N.Q.	0.7 ± 0.0	N.D.	N.D.	N.Q.	0.7 ± 0.0	3.3 ± 0.1	1.3 ± 0.0	0.6
7	B-type procyanidin dimer	N.D.	N.D.	0.9 ± 0.0	1.7 ± 0.0	0.4 ± 0.0	0.5 ± 0.0	0.5 ± 0.0	2.7 ± 0.1	6.8 ± 0.2	4.3 ± 0.1	1.8
12	(+) -Catechin	17.6 ± 0.3	16.5 ± 0.2	26.1 ± 0.4	40.1 ± 0.7	25.5 ± 0.4	12.9 ± 0.2	7.0 ± 0.1	26.6 ± 0.5	99.3 ± 1.3	134.3 ± 1.2	40.6
14	B-type procyanidin trimer	N.D.	N.D.	0.5 ± 0.0	1.6 ± 0.0	N.D.	N.D.	N.Q.	0.8 ± 0.0	3.2 ± 0.1	1.4 ± 0.0	0.7
17	B-type procyanidin dimer	N.D.	N.D.	2.2 ± 0.0	6.2 ± 0.1	1.1 ± 0.0	1.2 ± 0.0	1.9 ± 0.0	5.98 ± 0.1	37.8 ± 0.7	8.3 ± 0.2	6.5
18	B-type procyanidin trimer	N.D.	N.D.	N.D.	N.D.	N.D.	N.D.	N.D.	N.D.	1.9 ± 0.0	N.Q.	0.2
19	A-type procyanidin trimer	N.D.	N.D.	N.D.	N.D.	N.D.	N.D.	N.Q.	0.6 ± 0.0	1.6 ± 0.1	0.8 ± 0.0	0.3
24	(-)-Epicatechin	29.2 ± 0.5	18.3 ± 0.2	48.7 ± 0.9	85.7 ± 0.9	120.4 ± 1.7	40.0 ± 0.8	29.7 ± 0.4	69.8 ± 1.3	445.7 ± 7.2	219.6 ± 2.6	110.7
27	A-type procyanidin trimer	0.5 ± 0.0	N.D.	0.7 ± 0.0	0.7 ± 0.0	0.5 ± 0.0	N.Q.	N.Q.	0.9 ± 0.1	1.7 ± 0.0	2.3 ± 0.1	0.7
29	A-type procyanidin trimer	0.7 ± 0.0	N.D.	1.7 ± 0.0	2.0 ± 0.0	2.9 ± 0.1	1.5 ± 0.1	1.1 ± 0.0	3.4 ± 0.1	10.0 ± 0.1	5.5 ± 0.1	2.9
30	A-type procyanidin dimer	N.D.	N.D.	N.Q.	0.6 ± 0.0	N.D.	N.D.	N.Q.	0.9 ± 0.0	3.2 ± 0.1	1.2 ± 0.0	0.6
33	B-type procyanidin trimer	N.D.	N.D.	0.6 ± 0.0	3.0 ± 0.0	N.D.	N.D.	0.6 ± 0.0	2.0 ± 0.0	15.2 ± 0.1	2.3 ± 0.0	2.4
35	B-type procyanidin dimer	N.D.	N.D.	N.Q.	0.9 ± 0.0	N.D.	N.D.	N.Q.	0.6 ± 0.0	2.1 ± 0.1	1.9 ± 0.1	0.6
37	A-type procyanidin dimer	0.5 ± 0.0	N.D.	N.Q.	1.0 ± 0.0	1.5 ± 0.0	N.Q.	1.2 ± 0.0	0.7 ± 0.0	4.0 ± 0.1	2.0 ± 0.1	1.1
40	B-type procyanidin tetramer	N.D.	N.D.	N.D.	0.7 ± 0.0	N.D.	N.D.	N.D.	0.4 ± 0.0	2.7 ± 0.0	0.7 ± 0.0	0.5
44	A-type procyanidin trimer	N.D.	N.D.	0.7 ± 0.0	1.0 ± 0.0	N.D.	N.D.	N.Q.	1.1 ± 0.0	2.9 ± 0.1	2.1 ± 0.1	0.8
45	A-type procyanidin dimer	2.7 ± 0.1	2.5 ± 0.1	2.1 ± 0.0	1.7 ± 0.0	3.0 ± 0.1	1.7 ± 0.0	1.1 ± 0.0	4.5 ± 0.1	3.6 ± 0.1	7.4 ± 0.1	3.0
53	B-type procyanidin trimer	N.D.	N.D.	N.D.	N.D.	N.D.	N.D.	N.D.	N.D.	1.1 ± 0.0	N.Q.	0.1
60	B-type procyanidin dimer	N.D.	N.D.	0.9 ± 0.1	2.3 ± 0.0	1.0 ± 0.0	0.6 ± 0.0	0.7 ± 0.0	1.7 ± 0.0	10.2 ± 0.3	3.2 ± 0.0	2.1
63	B-type procyanidin trimer	N.D.	N.D.	N.D.	0.6 ± 0.0	N.D.	N.D.	N.D.	N.D.	2.1 ± 0.1	0.5 ± 0.0	0.3
66	A-type procyanidin dimer	3.6 ± 0.1	2.1 ± 0.0	4.5 ± 0.0	5.2 ± 0.1	10.6 ± 0.2	4.8 ± 0.1	3.6 ± 0.1	8.6 ± 0.2	20.1 ± 0.2	14.4 ± 0.3	7.8
67	A-type procyanidin trimer	2.0 ± 0.0	1.7 ± 0.0	1.6 ± 0.1	0.8 ± 0.0	1.9 ± 0.0	1.0 ± 0.0	N.Q.	1.5 ± 0.0	1.4 ± 0.0	5.2 ± 0.1	1.7
70	A-type procyanidin trimer	N.D.	N.D.	1.0 ± 0.0	1.8 ± 0.0	1.3 ± 0.0	0.9 ± 0.0	0.8 ± 0.0	2.6 ± 0.0	8.9 ± 0.1	4.3 ± 0.0	2.2
84	A-type procyanidin dimer	2.9 ± 0.1	2.8 ± 0.1	3.1 ± 0.1	3.7 ± 0.0	5.7 ± 0.1	3.2 ± 0.0	2.3 ± 0.0	5.6 ± 0.1	16.1 ± 0.2	10.8 ± 0.1	5.6
91	A-type procyanidin trimer	3.6 ± 0.1	2.2 ± 0.1	2.9 ± 0.1	1.7 ± 0.0	10.6 ± 0.3	3.9 ± 0.1	1.9 ± 0.1	4.4 ± 0.1	6.7 ± 0.2	7.8 ± 0.2	4.6
Total flavan-3-ols		63.5 ± 0.1	46.1 ± 0.5	98.0 ± 0.4	163.8 ± 0.9	186.3 ± 2.3	72.1 ± 0.8	52.3 ± 0.6	146.1 ± 1.6	711.7 ± 8.5	441.2 ± 3.8	198.1
Total polyphenols (mg/g FW)		17.9 ± 0.1	19.8 ± 0.1	16.3 ± 0.1	15.9 ± 0.2	21.4 ± 0.1	19.2 ± 0.1	11.8 ± 0.1	11.4 ± 0.1	12.9 ± 0.1	17.2 ± 0.1	16.4
***Triterpenoid***												
1′	Euscaphic acid	N.D.	N.D.	N.D.	0.4 ± 0.0	0.3 ± 0.0	N.D.	0.5 ± 0.0	N.D.	N.D.	N.D.	0.1
2′	Tormentic acid	6.0 ± 0.1	20.5 ± 0.5	14.5 ± 0.4	13.4 ± 0.3	11.6 ± 0.3	8.2 ± 0.2	5.3 ± 0.1	7.6 ± 0.1	15.8 ± 0.2	13.1 ± 0.2	11.6
3′	Anmurcoic acid	13.9 ± 0.2	55.3 ± 0.7	9.6 ± 0.1	18.0 ± 0.4	11.1 ± 0.1	8.7 ± 0.2	11.0 ± 0.2	6.7 ± 0.1	3.8 ± 0.1	9.3 ± 0.1	14.8
4′	Pomolic acid isomer	82.2 ± 1.2	60.7 ± 1.0	15.0 ± 0.3	25.5 ± 0.3	42.1 ± 0.8	2.9 ± 0.1	64.5 ± 1.2	50.0 ± 0.9	9.5 ± 0.2	81.1 ± 1.1	43.4
5′	1-Hydroxy-3-oxours-12-en-28-oic acid/isomer	4.6 ± 0.1	7.3 ± 0.1	N.D.	0.5 ± 0.0	0.4 ± 0.0	N.D.	1.5 ± 0.0	2.3 ± 0.0	N.D.	1.2 ± 0.0	1.8
6′	Pomolic acid	6.6 ± 0.1	2.4 ± 0.0	1.8 ± 0.1	2.6 ± 0.1	1.9 ± 0.1	5.9 ± 0.1	4.5 ± 0.1	1.9 ± 0.0	2.7 ± 0.1	9.7 ± 0.2	4.0
7′	701 *m/z* [M − H]^-^	6.1 ± 0.1	8.3 ± 0.2	5.2 ± 0.1	6.1 ± 0.2	5.3 ± 0.1	4.6 ± 0.1	4.9 ± 0.0	7.5 ± 0.2	13.4 ± 0.1	19.1 ± 0.3	8.1
8′	Alphitolic acid	1.4 ± 0.0	1.6 ± 0.0	0.4 ± 0.0	1.1 ± 0.0	0.9 ± 0.0	2.3 ± 0.0	2.7 ± 0.1	0.4 ± 0.0	0.9 ± 0.0	0.5 ± 0.0	1.2
9′	Maslinic acid	4.0 ± 0.1	28.5 ± 0.4	4.5 ± 0.1	10.9 ± 0.3	10.7 ± 0.2	11.1 ± 0.2	6.7 ± 0.1	7.8 ± 0.1	9.7 ± 0.1	8.7 ± 0.2	10.2
10′	Corosolic acid	2.3 ± 0.1	9.8 ± 0.2	5.2 ± 0.1	11.9 ± 0.2	8.8 ± 0.2	35.7 ± 0.5	2.8 ± 0.1	5.2 ± 0.1	28.4 ± 0.5	5.2 ± 0.1	11.5
11′	1-Hydroxy-3-oxours-12-en-28-oic acid/isomer	N.D.	0.9 ± 0.0	N.D.	N.D.	N.D.	N.D.	N.D.	N.D.	N.D.	N.D.	0.1
12′	1-Hydroxy-3-oxours-12-en-28-oic acid/isomer	N.D.	0.9 ± 0.0	1.3 ± 0.0	2.1 ± 0.0	N.D.	0.9 ± 0.0	N.D.	N.D.	0.3 ± 0.0	N.D.	0.5
13′	Betulinic acid	8.1 ± 0.1	1.4 ± 0.0	1.2 ± 0.0	2.3 ± 0.0	2.6 ± 0.0	6.2 ± 0.1	13.0 ± 0.1	1.8 ± 0.0	0.6 ± 0.0	1.6 ± 0.0	3.9
14′	Oleanolic acid	38.5 ± 0.6	37.1 ± 0.4	35.7 ± 0.4	37.2 ± 0.5	38.5 ± 0.4	49.9 ± 0.6	49.0 ± 0.7	37.8 ± 0.6	13.5 ± 0.3	39.6 ± 0.5	37.7
15′	Ursolic acid	113.1 ± 1.7	112.8 ± 1.1	149.7 ± 1.6	146.5 ± 1.8	129.3 ± 1.3	257.1 ± 2.3	97.1 ± 1.98	125.5 ± 1.8	66.7 ± 1.1	94.9 ± 1.2	129.3
16′	687 *m*/*z* [M − H]^-^	18.9 ± 0.3	20.1 ± 0.4	16.1 ± 0.2	19.0 ± 0.2	11.0 ± 0.2	9.8 ± 0.2	14.2 ± 0.2	19.8 ± 0.2	28.0 ± 0.5	49.3 ± 0.6	20.6
Total triterpenoids		305.7 ± 1.12	367.5 ± 1.4	260.3 ± 1.1	297.4 ± 1.4	274.6 ± 2.6	403.1 ± 2.2	277.5 ± 3.4	274.3 ± 3.2	193.0 ± 1.2	333.3 ± 2.6	298.7

Note: N.D., not detectable; N.Q., not quantifiable; DSSL, ‘Dangshansuli’; YL, ‘Yali’; CG, ‘Cuiguan’; FS, ‘Fengshui’; NGL, ‘Nanguoli’; XSL, ‘Xiangshuili’; KELXL, ‘Kuerlexiangli’; XJSL, ‘Xinjiangsuanli’; HQL, ‘Hongqieli’; SJL, ‘Sanjili’.

**Table 4:** Antioxidant capacities of thinned young pears based on DPPH analysis (mean ± SD, *n* = 3).

Pear Variety	Antioxidant Capacity (μmol TE/g FW)	Pear Variety	Antioxidant Capacity (μmol TE/g FW)
DSSL	20.7 ± 0.2 ^c^	KELXL	10.1 ± 0.2 ^g^
YL	20.6 ± 0.2 ^c^	XJSL	22.5 ± 0.3 ^b^
CG	12.4 ± 0.2 ^f^	HQL	30.4 ± 0.3 ^a^
FS	15.6 ± 0.1 ^e^	SJL	19.9 ± 0.2 ^d^
NGL	22.2 ± 0.2 ^b^	mean	19.7
XSL	22.4 ± 0.2 ^b^	C.V. (%)	29.4

Note: different letters indicate significantly different at *p* < 0.05.

## Supplementary Materials

The following are available online, Figure S1: Representative UPLC-Q TRAP-MS/MS total ion chromatograms of thinned young pears in negative ion mode. Figure S2: Representative MRM chromatograms for 102 polyphenols and 16 triterpenoids in thinned pears. Table S1: The optimized MS parameters at MRM mode and summary of the intra- and inter-day precision for available analytes. Table S2: Repeatability and stability for 102 polyphenols in representative pear extracts. Table S3: Repeatability and stability for 16 triterpenoids in representative pear extracts. Table S4: The correlation coefficients between antioxidant activity and chemical compounds in thinned young pears.
