# Supplementary material for: Characterization and Quantification of Polyphenols and Triterpenoids in Thinned Young Fruits of Ten Pear Varieties by UPLC-Q TRAP-MS/MS
Source: Molecules. 2019 Jan 3;24(1):159. doi: 10.3390/molecules24010159 (PMC6337724; doi:10.3390/molecules24010159)
Supplement: Supplementary file 1 [file molecules-24-00159-s001.pdf]

molecules

Article

Characterization and quantification of polyphenols and triterpenoids in thinned young fruits of ten pear varieties by UPLC-Q TRAP-MS/MS

Liqiong Sun, Shutian Tao and Shaoling Zhang *

Center of Pear Engineering Technology Research, State Key Laboratory of Crop Genetics and Germplasm Enhancement, Nanjing Agricultural University, Nanjing 210095, China; sarlign@163.com (L.S.); taost@njau.edu.cn (S.T.)

* Correspondence: slzhang@njau.edu.cn; Tel.: +86-25-84396580

(Supplementary Material)

Figure S1: Representative UPLC-Q TRAP-MS/MS total ion chromatogram of thinned young pears in negative ion mode. (A) the polyphenolic profiles of 'Hongqieli' extract, (B) the triterpenoid profiles of 'Yali' extract.

Figure S2: Representative MRM chromatograms for 102 polyphenols and 16 triterpenoids in thinned pears.

Table S1: The optimized MS parameters at MRM mode and summary of the intra- and inter-day precision for available analytes.

Table S2: Repeatability and stability for 102 polyphenols in representative pear extracts.

Table S3: Repeatability and stability for 16 triterpenoids in representative pear extracts.

Table S4: The correlation coefficients between antioxidant activity and chemical compounds in thinned young pears.

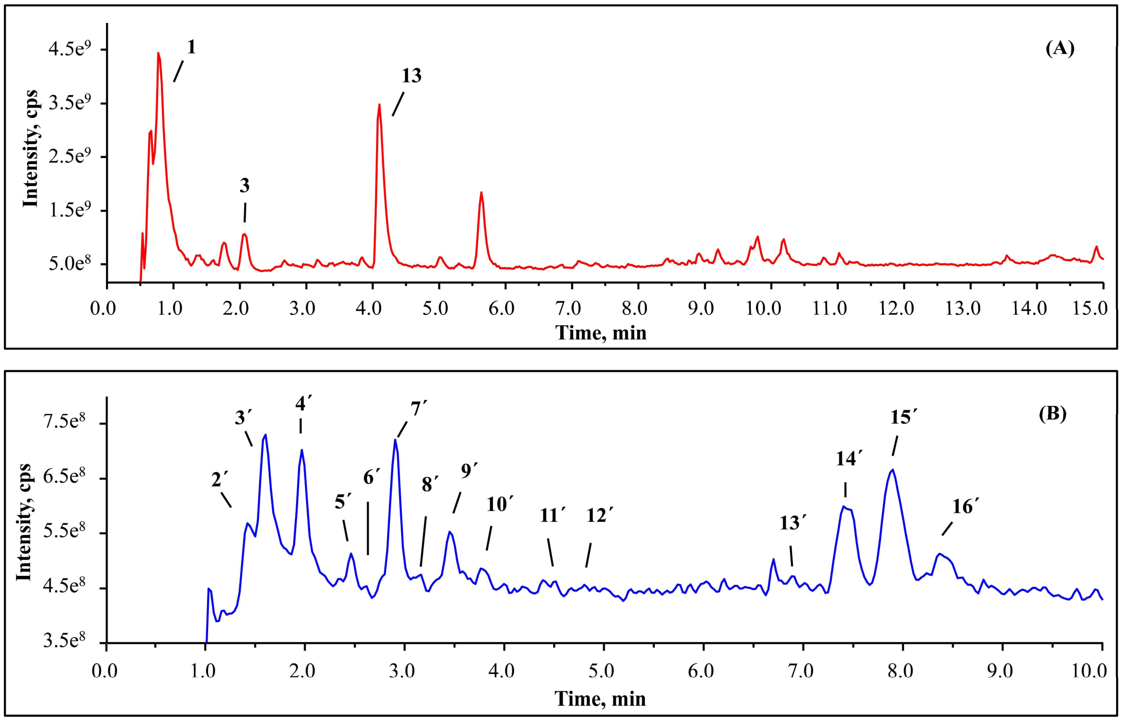

Figure 1. Representative UPLC-Q TRAP-MS/MS total ion chromatogram of thinned young pears in negative ion mode. (**A**) the polyphenolic profiles of 'Hongqieli' extract, (**B**) the triterpenoid profiles of 'Yali' extract.

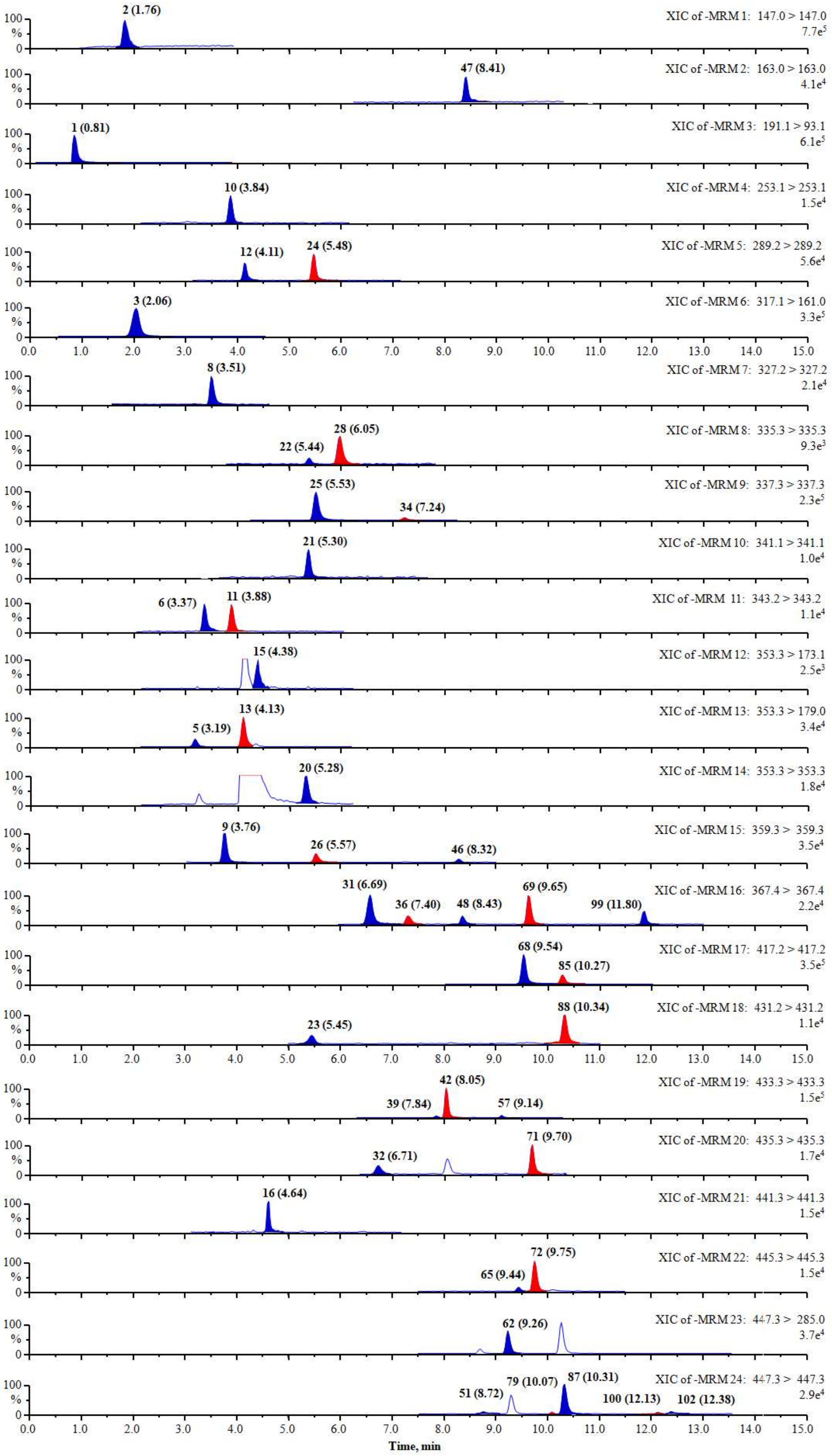
2 (1.76)
XIC of -MRM 1: 147.0 > 147.0
7.7e5
47 (8.41)
XIC of -MRM 2: 163.0 > 163.0
4.1e4
1 (0.81)
XIC of -MRM 3: 191.1 > 93.1
6.1e5
10 (3.84)
XIC of -MRM 4: 253.1 > 253.1
1.5e4
12 (4.11)
24 (5.48)
XIC of -MRM 5: 289.2 > 289.2
5.6e4
3 (2.06)
XIC of -MRM 6: 317.1 > 161.0
3.3e5
8 (3.51)
XIC of -MRM 7: 327.2 > 327.2
2.1e4
22 (5.44)
28 (6.05)
XIC of -MRM 8: 335.3 > 335.3
9.3e3
25 (5.53)
34 (7.24)
XIC of -MRM 9: 337.3 > 337.3
2.3e5
21 (5.30)
XIC of -MRM 10: 341.1 > 341.1
1.0e4
6 (3.37)
11 (3.88)
XIC of -MRM 11: 343.2 > 343.2
1.1e4
15 (4.38)
XIC of -MRM 12: 353.3 > 173.1
2.5e3
5 (3.19)
13 (4.13)
XIC of -MRM 13: 353.3 > 179.0
3.4e4
20 (5.28)
XIC of -MRM 14: 353.3 > 353.3
1.8e4
9 (3.76)
26 (5.57)
46 (8.32)
XIC of -MRM 15: 359.3 > 359.3
3.5e4
31 (6.69)
36 (7.40)
48 (8.43)
69 (9.65)
99 (11.80)
XIC of -MRM 16: 367.4 > 367.4
2.2e4
68 (9.54)
85 (10.27)
XIC of -MRM 17: 417.2 > 417.2
3.5e5
23 (5.45)
88 (10.34)
XIC of -MRM 18: 431.2 > 431.2
1.1e4
39 (7.84)
42 (8.05)
57 (9.14)
XIC of -MRM 19: 433.3 > 433.3
1.5e5
32 (6.71)
71 (9.70)
XIC of -MRM 20: 435.3 > 435.3
1.7e4
16 (4.64)
XIC of -MRM 21: 441.3 > 441.3
1.5e4
65 (9.44)
72 (9.75)
XIC of -MRM 22: 445.3 > 445.3
1.5e4
62 (9.26)
XIC of -MRM 23: 447.3 > 285.0
3.7e4
51 (8.72)
79 (10.07)
87 (10.31)
100 (12.13)
102 (12.38)
XIC of -MRM 24: 447.3 > 447.3
2.9e4
100
%
0
0.0 1.0 2.0 3.0 4.0 5.0 6.0 7.0 8.0 9.0 10.0 11.0 12.0 13.0 14.0 15.0
Time, min

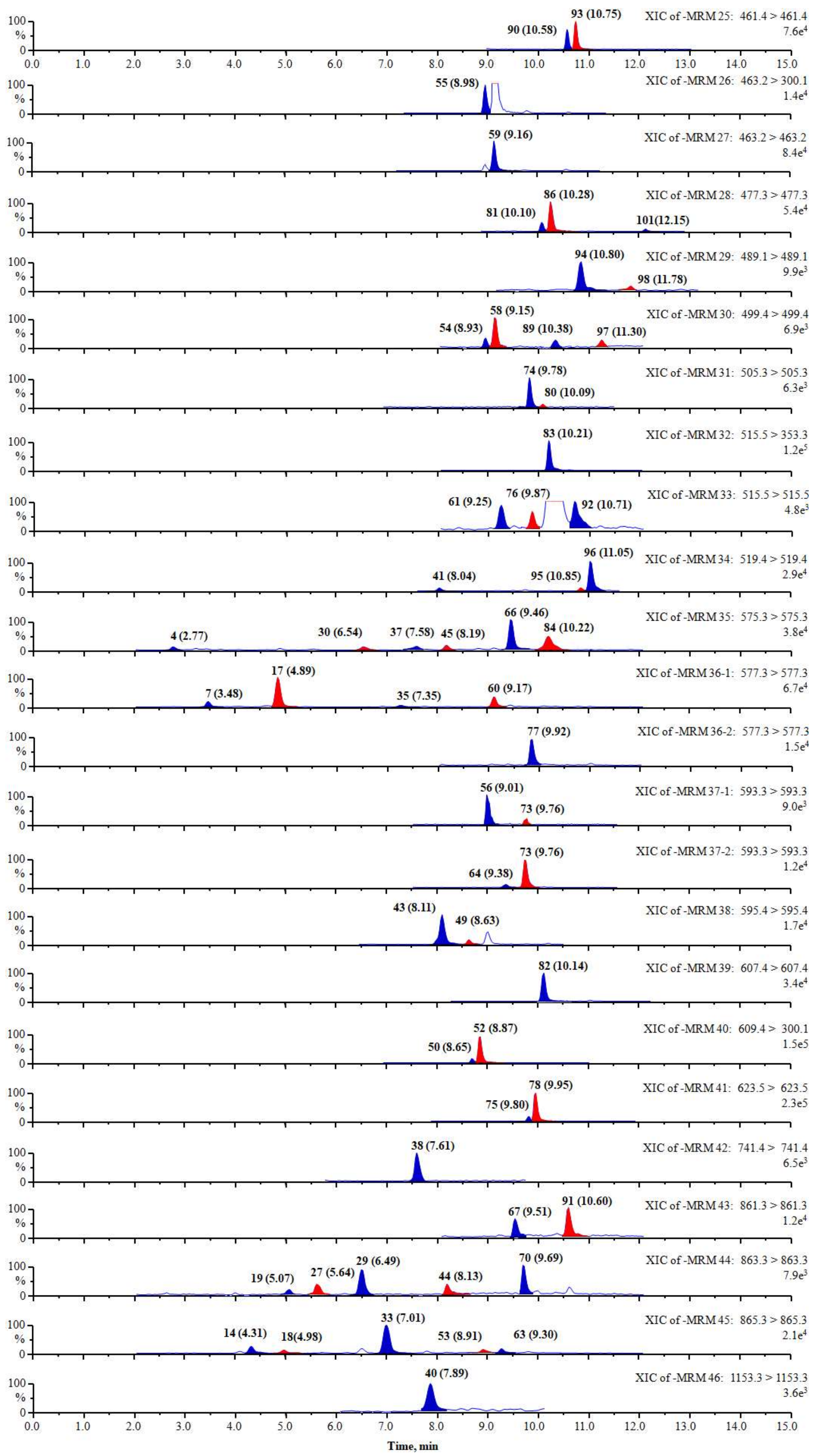
XIC of -MRM 25: 461.4 > 461.4
7.6e4
90 (10.58)
93 (10.75)
XIC of -MRM 26: 463.2 > 300.1
1.4e4
55 (8.98)
XIC of -MRM 27: 463.2 > 463.2
8.4e4
59 (9.16)
XIC of -MRM 28: 477.3 > 477.3
5.4e4
81 (10.10)
86 (10.28)
101(12.15)
XIC of -MRM 29: 489.1 > 489.1
9.9e3
94 (10.80)
98 (11.78)
XIC of -MRM 30: 499.4 > 499.4
6.9e3
54 (8.93)
58 (9.15)
89 (10.38)
97 (11.30)
XIC of -MRM 31: 505.3 > 505.3
6.3e3
74 (9.78)
80 (10.09)
XIC of -MRM 32: 515.5 > 353.3
1.2e5
83 (10.21)
XIC of -MRM 33: 515.5 > 515.5
4.8e3
61 (9.25)
76 (9.87)
92 (10.71)
XIC of -MRM 34: 519.4 > 519.4
2.9e4
41 (8.04)
95 (10.85)
96 (11.05)
XIC of -MRM 35: 575.3 > 575.3
3.8e4
4 (2.77)
30 (6.54)
37 (7.58)
45 (8.19)
66 (9.46)
84 (10.22)
XIC of -MRM 36-1: 577.3 > 577.3
6.7e4
7 (3.48)
17 (4.89)
35 (7.35)
60 (9.17)
XIC of -MRM 36-2: 577.3 > 577.3
1.5e4
77 (9.92)
XIC of -MRM 37-1: 593.3 > 593.3
9.0e3
56 (9.01)
73 (9.76)
XIC of -MRM 37-2: 593.3 > 593.3
1.2e4
64 (9.38)
73 (9.76)
XIC of -MRM 38: 595.4 > 595.4
1.7e4
43 (8.11)
49 (8.63)
XIC of -MRM 39: 607.4 > 607.4
3.4e4
82 (10.14)
XIC of -MRM 40: 609.4 > 300.1
1.5e5
50 (8.65)
52 (8.87)
XIC of -MRM 41: 623.5 > 623.5
2.3e5
75 (9.80)
78 (9.95)
XIC of -MRM 42: 741.4 > 741.4
6.5e3
38 (7.61)
XIC of -MRM 43: 861.3 > 861.3
1.2e4
67 (9.51)
91 (10.60)
XIC of -MRM 44: 863.3 > 863.3
7.9e3
19 (5.07)
27 (5.64)
29 (6.49)
44 (8.13)
70 (9.69)
XIC of -MRM 45: 865.3 > 865.3
2.1e4
14 (4.31)
18(4.98)
33 (7.01)
53 (8.91)
63 (9.30)
XIC of -MRM 46: 1153.3 > 1153.3
3.6e3
40 (7.89)
100
%
0
0.0 1.0 2.0 3.0 4.0 5.0 6.0 7.0 8.0 9.0 10.0 11.0 12.0 13.0 14.0 15.0
Time, min

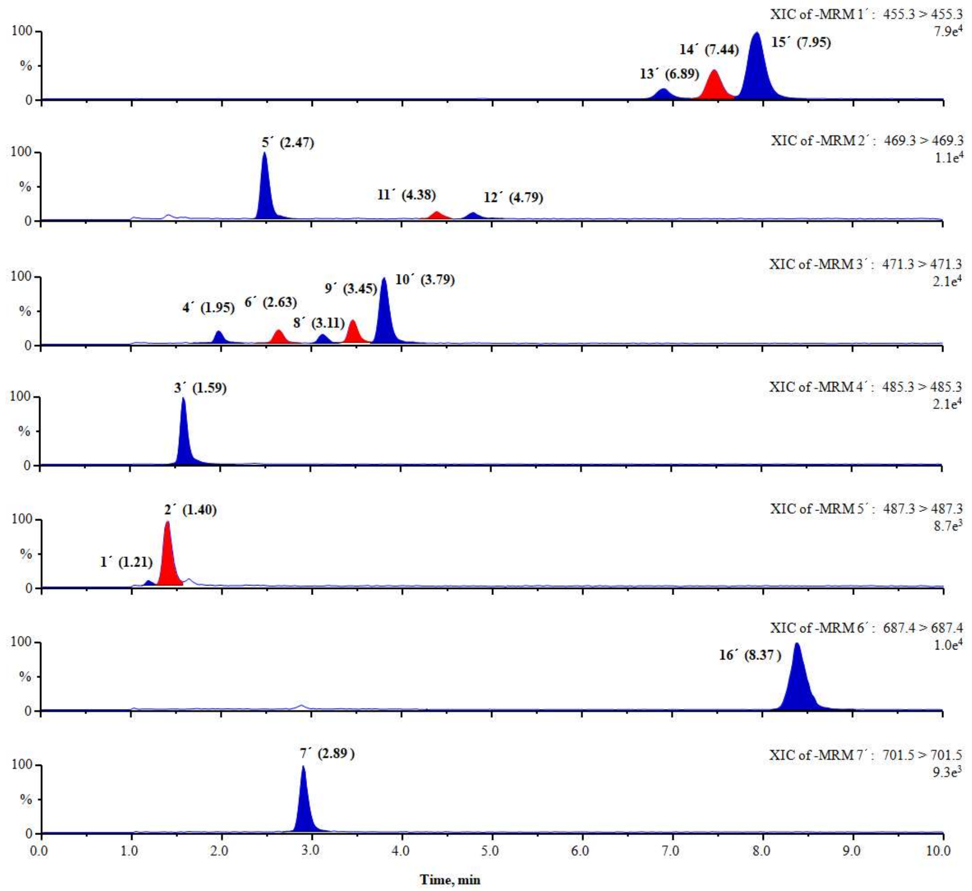

Figure 2. Representative MRM chromatograms for 102 polyphenols and 16 triterpenoids in thinned pears.

Table 1. The optimized MS parameters at MRM mode and summary of the intra- and inter-day precision for available analytes.

Compound	MS parameters			Precision (%)	
	Ion transition	DP (V)	CE (eV)	Intra-day	Inter-day
Quinic acid	191 > 93	-105	-30	0.97	1.96
Arbutin	317 > 161	-40	-20	0.87	2.46
(+) -Catechin	289 > 289	-90	-8	0.74	2.21
(-)-Epicatechin	289 > 289	-90	-8	0.44	2.56
Chlorogenic acid	353 > 179	-75	-25	0.93	2.14
Cryptochlorogenic acid	353 > 179	-75	-25	1.09	2.54
Neochlorogenic acid	353 > 173	-75	-25	0.83	3.19
Isochlorogenic acid A	515 > 353	-90	-25	0.77	3.26
Isochlorogenic acid B	515 > 353	-90	-25	0.82	2.13
Isochlorogenic acid C	515 > 353	-90	-25	0.43	2.47
p-Coumaric acid	163 > 163	-60	-8	1.12	1.64
Caffeic acid	179 > 179	-70	-8	1.07	2.56
Rutin	609 > 300	-150	-50	0.85	2.13
	609 > 609	-150	-8	0.64	2.78
Kaempferol-3-*O*-rutinoside	593 > 285	-150	-43	1.38	2.82
	593 > 593	-150	-8	1.09	2.33
Isorhamnetin-3-*O*-rutinoside	623 > 315	-135	-43	1.25	2.94
	623 > 623	-135	-8	1.62	2.81

Luteoloside	447 > 285	-135	-40	0.71	1.47
	447 > 447	-135	-8	0.94	2.55
Hyperoside	463 > 300	-115	-38	0.83	2.07
Oleanic acid	455 > 455	-150	-10	0.75	1.84
Ursolic acid	455 > 455	-150	-10	0.68	2.40
Betulinic acid	455 > 455	-150	-10	0.63	2.08
Pomolic acid	471 > 471	-150	-10	0.95	2.11
Maslinic acid	471 > 471	-150	-10	0.82	2.59
Corosolic acid	471 > 471	-150	-10	1.13	2.86

Table 2. Repeatability and stability for 102 polyphenols in representative pear extracts.

Peak NO.	Repeatability (%)				Stability (%)			
	DSSL	NGL	HQL	SJL	DSSL	NGL	HQL	SJL
1	2.87	2.14	2.05	1.69	1.97	1.29	1.62	2.06
2	2.29	2.37	1.75	2.50	2.82	1.64	1.33	1.01
3	2.58	2.16	2.50	2.71	1.86	1.73	1.91	1.60
4	-	-	4.90	3.06	-	-	3.24	2.47
5	3.49	2.09	3.30	2.50	1.84	2.39	2.71	3.09
6	2.68	2.75	2.40	3.20	1.74	1.99	1.29	2.74
7	-	5.19	2.34	3.39	-	3.38	1.69	2.11
8	2.38	4.45	6.26	5.97	3.59	3.74	4.95	3.92
9	3.18	-	3.11	2.82	2.75	-	3.29	2.59
10	2.47	-	2.13	2.45	1.89	-	3.86	2.38
11	2.86	3.48	2.07	3.28	2.25	1.88	2.22	2.35
12	2.42	2.30	2.94	2.55	2.18	2.16	1.56	2.47
13	2.45	3.99	2.83	2.62	2.31	3.35	2.73	2.05
14	-	-	4.72	3.45	-	-	2.59	2.39
15	2.55	2.72	3.08	3.26	2.40	1.56	1.90	2.80
16	-	-	2.50	3.17	-	-	2.21	1.67
17	-	3.30	2.86	2.26	-	2.02	1.66	2.89
18	-	-	2.51	-	-	-	2.20	-
19	-	-	4.86	5.96	-	-	3.37	4.80
20	2.97	3.51	2.81	2.82	2.47	2.34	2.07	2.59
21	3.26	1.72	2.45	2.65	1.42	2.68	1.43	2.90
22	3.48	4.26	4.18	3.67	2.29	3.07	2.02	3.05
23	4.91	5.18	5.29	3.12	3.93	3.84	4.67	2.95
24	2.65	3.07	2.23	2.55	1.27	2.42	1.68	2.30
25	3.34	2.14	2.90	2.91	1.97	2.54	2.58	1.68
26	2.29	3.62	3.84	4.20	2.30	2.51	1.86	2.47
27	4.51	5.74	2.63	3.14	1.97	2.32	1.62	1.95
28	4.04	2.12	3.89	2.73	2.27	1.37	3.64	2.05
29	3.38	2.14	2.98	2.39	2.72	3.33	2.34	1.93
30	-	-	3.78	2.93	-	-	2.95	2.47
31	2.26	2.60	3.31	2.05	1.80	2.44	1.82	1.59
32	2.28	3.01	3.14	1.66	2.56	1.90	2.18	2.51
33	-	-	2.54	3.02	-	-	1.77	2.21
34	2.93	4.71	3.21	3.17	2.45	4.07	2.90	2.71
35	-	-	2.67	3.37	-	-	2.22	1.63
36	2.87	3.34	3.02	2.98	1.98	1.68	1.29	2.50
37	5.09	6.87	3.32	5.17	3.38	4.73	3.99	2.77
38	4.71	5.74	-	-	3.95	2.80	-	-
39	-	-	-	2.04	-	-	-	1.77

40	-	-	4.10	5.68	-	-	2.92	3.65
41	3.87	-	-	-	3.30	-	-	-
42	2.39	-	-	2.82	1.68	-	-	1.47
43	2.19	3.09	-	-	2.76	3.62	-	-
44	-	-	3.64	5.87	-	-	2.25	1.87
45	3.26	2.47	2.80	3.47	3.23	2.85	2.41	2.40
46	4.84	4.46	-	7.65	4.56	4.35	-	4.48
47	2.74	3.00	2.58	3.06	2.11	2.36	1.63	1.87
48	-	-	2.97	2.75	-	-	2.31	1.92
49	-	-	3.69	4.07	-	-	2.09	3.67
50	4.88	4.10	2.67	3.08	4.12	5.24	3.20	2.74
51	-	4.71	-	-	-	4.22	-	-
52	3.61	3.11	2.80	2.76	1.70	2.58	1.53	1.41
53	-	-	4.47	-	-	-	1.94	-
54	-	3.44	-	-	-	2.35	-	-
55	3.58	4.07	5.20	3.33	1.67	4.19	2.29	1.79
56	2.25	3.90	-	-	1.65	2.84	-	-
57	2.02	-	-	3.41	1.93	-	-	2.63
58	-	3.82	-	-	-	2.21	-	-
59	2.53	3.13	4.29	2.62	2.67	1.97	1.96	2.65
60	-	2.36	3.95	3.44	-	1.50	2.34	2.40
61	2.30	3.28	-	-	2.31	2.79	-	-
62	2.40	3.03	-	-	1.42	2.36	-	-
63	-	-	2.74	4.59	-	-	1.82	2.70
64	-	-	4.42	6.25	-	-	2.21	3.33
65	-	3.99	5.73	4.54	-	2.49	3.84	3.27
66	2.36	3.59	2.41	3.18	2.11	2.88	1.72	2.27
67	2.27	3.49	4.15	2.53	2.29	1.16	3.10	2.46
68	1.54	4.35	6.68	6.53	1.39	4.47	3.08	5.37
69	1.60	2.56	3.14	2.47	2.29	2.17	2.39	1.88
70	-	2.94	2.69	3.83	-	2.19	1.34	2.61
71	1.75	2.51	2.70	3.12	2.41	3.19	2.09	2.53
72	4.55	-	4.70	4.62	3.23	-	2.18	2.20
73	4.33	5.31	2.81	3.60	3.31	2.05	2.53	2.18
74	1.67	4.86	2.55	2.52	1.07	2.28	2.32	1.80
75	2.00	1.99	2.73	2.77	1.61	2.76	1.85	1.28
76	4.94	-	5.17	-	3.39	-	3.84	-
77	4.74	3.83	-	4.10	1.41	1.32	-	2.66
78	2.18	3.81	3.32	4.08	1.52	2.91	1.72	1.02
79	3.33	4.88	2.60	3.36	2.25	2.92	2.64	2.34
80	3.92	4.84	2.54	2.98	1.86	4.29	1.09	1.66
81	3.79	5.42	4.75	4.16	1.79	3.83	1.77	1.46
82	2.65	3.47	-	-	3.03	1.60	-	-
83	3.08	3.51	3.70	3.93	2.26	2.58	2.55	2.49
84	1.71	2.87	2.54	1.31	1.47	1.96	1.00	1.11
85	4.38	4.99	3.36	3.52	2.18	4.05	3.17	1.99
86	3.40	4.86	3.65	3.97	1.21	1.28	2.56	2.83
87	4.67	2.79	-	-	1.45	1.89	-	-
88	2.39	3.06	5.79	4.11	2.13	2.45	2.29	1.99
89	5.26	3.70	4.61	5.49	2.48	2.34	2.39	3.87
90	2.25	1.61	-	-	1.85	1.02	-	-
91	3.33	2.57	2.74	2.19	2.32	1.48	3.08	1.57

92	2.94	2.51	3.95	4.09	2.03	2.26	1.70	2.08
93	5.94	2.71	5.92	6.27	4.07	1.31	3.79	3.78
94	3.18	4.74	3.10	2.34	1.29	1.24	2.59	1.04
95	3.69	-	2.76	3.28	1.23	-	2.64	1.01
96	2.92	2.91	2.97	3.43	1.75	2.98	1.84	2.84
97	3.27	5.22	3.00	2.24	2.36	3.19	3.62	2.44
98	4.28	-	-	5.85	3.40	-	-	5.17
99	3.22	2.96	3.24	3.61	1.52	1.79	2.13	2.27
100	5.46	5.43	6.39	6.86	3.03	5.71	4.46	3.25
101	3.59	-	-	-	1.99	-	-	-
102	6.30	5.16	7.29	5.57	6.74	5.15	5.23	5.16

Table 3. Repeatability and stability for 16 triterpenoids in representative pear extracts.

Peak NO.	Repeatability (%)		Stability (%)	
	YL	NGL	YL	NGL
1′	-	5.65	-	2.37
2′	3.04	3.46	1.94	2.15
3′	3.74	4.14	1.40	2.01
4′	2.70	2.11	2.60	2.09
5′	2.84	4.03	2.16	2.99
6′	4.02	3.93	1.61	1.83
7′	3.06	4.18	1.45	1.98
8′	3.31	4.43	1.12	1.84
9′	3.53	3.91	2.48	2.06
10′	3.86	4.12	2.79	2.51
11′	4.05	-	1.71	-
12′	4.58	-	1.08	-
13′	3.99	2.85	1.78	1.92
14′	2.91	2.10	2.28	1.14
15′	2.15	2.59	1.73	1.35
16′	2.50	3.53	1.62	1.73

Table 4. The correlation coefficients between antioxidant activity and chemical compounds in thinned young pears.

Peak NO.	Compound	Correlation coefficient	Peak NO.	Compound	Correlation coefficient
	Phenolic acid			***Phenolic glycoside***	
1	Quinic acid	−0.265	3	Arbutin	0.013
2	Cinnamic acid isomer	0.322	6	Dihydro-caffeoyl-*O*-hexoside	0.193
5	Cryptochlorogenic acid	0.187	8	Hydroxyphenylpropionic acid-*O*-hexoside	0.153
10	Caffeoylglycerol	0.036	9	Syringic acid-*O*-hexoside	0.015
13	Chlorogenic acid	0.566	11	Dihydro-caffeoyl-*O*-hexoside	-0.429
15	Neochlorogenic acid	0.401	21	Caffeoyl-*O*-hexoside	0.079
16	*p*-Coumaroylcaffeoyl malate	0.607	23	Roseoside	0.352
20	1-*O*-Caffeoylquinic acid	0.784	26	Syringic acid-*O*-hexoside	0.780
22	Caffeoylshikimic acid	−0.103	39	Caffeoylarbutin	0.043
25	4-*p*-Coumaroylquinic acid	0.752	42	Caffeoylarbutin	-0.723
28	Caffeoylshikimic acid	−0.276	46	Syringic acid-*O*-hexoside	-0.156

31	3-*O*-Feruloylquinic acid	0.400
32	Caffeoyl-malonyl-methylcitric acid	0.398
34	5-*p*-Coumaroylquinic acid	0.671
36	4-*O*-Caffeoylquinic acid methyl ester	0.509
47	Hydroxycinnamic acid	0.460
48	3-*O*-Caffeoylquinic acid methyl ester	0.495
54	*p*-Coumaroylcaffeoylquinic acid	−0.095
58	*p*-Coumaroylcaffeoylquinic acid	−0.117
61	Di-*O*-caffeoylquinic acid	−0.308
69	5-*O*-Caffeoylquinic acid methyl ester	0.196
71	Caffeoyl-malonyl-methylcitric acid	0.209
76	Isochlorogenic acid B	0.454
83	Isochlorogenic acid A	0.227
89	*p*-Coumaroylcaffeoylquinic acid	0.588
92	Isochlorogenic acid C	−0.265
97	*p*-Coumaroylcaffeoylquinic acid	0.371
99	1-*O*-Caffeoylquinic acid methyl ester	0.356
	Flavone	
38	Quercetin-3-*O*-xylosylrhamnosylglucoside	-0.737
41	Isorhamnetin-acylated-hexoside	-0.689
43	Quercetin-3-*O*-arabinosylgalactoside	-0.577
49	Quercetin-3-*O*-arabinosylglucoside	0.576
50	Quercetin-3-*O*-rhamnosylgalactoside	0.641
51	Luteolin-7-*O*-galactoside	0.154
52	Rutin	0.379
55	Hyperoside	0.610
56	Kaempferol-3-*O*-rhamnosylgalactoside	−0.070
59	Quercetin-3-*O*-glucoside	0.547
62	Luteoloside	−0.286
64	Kaempferol-3-*O*-rhamnosylglucoside	0.576
65	Apigenin-*O*-glucuronide or isomer	0.615
72	Apigenin-*O*-glucuronide or isomer	−0.322

57	Caffeoylarbutin	-0.766
68	*p*-Coumaroylarbutin	-0.712
85	*p*-Coumaroylarbutin	-0.737
100	Galloyl-coumaric acid pentoside	0.444
102	Galloyl-coumaric acid pentoside	0.608
	Flavan-3-ol	
4	A-type procyanidin dimer	0.632
7	B-type procyanidin dimer	0.562
12	(+) -Catechin	0.404
14	B-type procyanidin trimer	0.492
17	B-type procyanidin dimer	0.625
18	B-type procyanidin trimer	0.651
19	A-type procyanidin trimer	0.675
24	(-)-Epicatechin	0.653
27	A-type procyanidin trimer	0.383
29	A-type procyanidin trimer	0.650
30	A-type procyanidin dimer	0.649
33	B-type procyanidin trimer	0.610
35	B-type procyanidin dimer	0.492
37	A-type procyanidin dimer	0.538
40	B-type procyanidin tetramer	0.630
44	A-type procyanidin trimer	0.488
45	A-type procyanidin dimer	0.394
53	B-type procyanidin trimer	0.651
60	B-type procyanidin dimer	0.612
63	B-type procyanidin trimer	0.576
66	A-type procyanidin dimer	0.669
67	A-type procyanidin trimer	0.244
70	A-type procyanidin trimer	0.619
84	A-type procyanidin dimer	0.688
91	A-type procyanidin trimer	0.550
	Triterpenoid	

73	Kaempferol-3-*O*-rutinoside	0.656	1′	Euscaphic acid	-0.525
74	Quercetin-acylated-galactoside	0.660	2′	Tormentic acid	0.238
75	Isorhamnetin-3-*O*-rhamnosylgalactoside	0.134	3′	Anmurcoic acid	-0.091
77	Apigenin rutinoside	0.117	4′	Pomolic acid or isomer	-0.205
78	Isorhamnetin-3-*O*-rutinoside	−0.460	5′	1-Hydroxy-3-oxours-12-en-28-oic acid or isomer	0.026
79	Kaempferol-3-*O*-galactoside	0.467	6′	Pomolic acid or isomer	0.001
80	Quercetin-acylated-glucoside	0.622	7′	701 *m*/*z* [M-H]$^-$	0.407
81	Isorhamnetin-3-*O*-galactoside	0.184	8′	Alphitolic acid	-0.274
82	Chrysoeriol-7-neohesperidoside	−0.017	9′	Maslinic acid	0.206
86	Isorhamnetin-3-*O*-glucoside	0.163	10′	Corosolic acid	0.570
87	Kaempferol-3-*O*-glucoside	0.126	11′	1-Hydroxy-3-oxours-12-en-28-oic acid or isomer	0.058
88	Apigenin-*O*-hexoside	0.096	12′	1-Hydroxy-3-oxours-12-en-28-oic acid or isomer	-0.321
90	Chrysoeriol-7-*O*-galactoside	−0.322	13′	Betulinic acid	-0.471
93	Chrysoeriol-7-*O*-glucoside	0.163	14′	Oleanolic acid	-0.614
94	Kaempferol-acylated-galactoside	0.664	15′	Ursolic acid	-0.108
95	Isorhamnetin-acylated-galactoside	0.332	16′	687 *m*/*z* [M-H]$^-$	0.218
96	Isorhamnetin-acylated-glucoside	0.312			
98	Kaempferol-acylated-glucoside	−0.483			
101	Phloretin-acylated-hexoside	−0.644			
